# Males induce premature demise of the opposite sex by multifaceted strategies

**DOI:** 10.1038/s43587-022-00276-y

**Published:** 2022-09-16

**Authors:** Lauren N. Booth, Cheng Shi, Cindy Tantilert, Robin W. Yeo, Jason W. Miklas, Katja Hebestreit, Cecilia N. Hollenhorst, Travis J. Maures, Matthew T. Buckley, Coleen T. Murphy, Anne Brunet

**Affiliations:** 1 Department of Genetics, Stanford University School of Medicine, Stanford, CA USA; 2 Department of Molecular Biology, Princeton University, Princeton, NJ USA; 3 LSI Genomics, Princeton University, Princeton, NJ USA; 4 Glenn Laboratories for the Biology of Aging and Stanford University, Stanford, CA USA; 5 Present Address: Calico Life Sciences, South San Francisco, CA USA

**Keywords:** Gene expression profiling, RNAi, Ageing, Metabolism

## Abstract

Interactions between the sexes negatively impact health in many species. In *Caenorhabditis*, males shorten the lifespan of the opposite sex—hermaphrodites or females. Here we use transcriptomic profiling and targeted screens to systematically uncover conserved genes involved in male-induced demise in *C.* *elegans*. Some genes (for example, *delm-2*, *acbp-3*), when knocked down, are specifically protective against male-induced demise. Others (for example, *sri-40*), when knocked down, extend lifespan with and without males, suggesting general mechanisms of protection. In contrast, many classical long-lived mutants are impacted more negatively than wild type by the presence of males, highlighting the importance of sexual environment for longevity. Interestingly, genes induced by males are triggered by specific male components (seminal fluid, sperm and pheromone), and manipulating these genes in combination in hermaphrodites induces stronger protection. One of these genes, the conserved ion channel *delm-2*, acts in the nervous system and intestine to regulate lipid metabolism. Our analysis reveals striking differences in longevity in single sex versus mixed sex environments and uncovers elaborate strategies elicited by sexual interactions that could extend to other species.

## Abstract (web summary)

Sexual interactions with males shorten the lifespan of the opposite sex in several species, including *Caenorhabditis elegans*, but the mechanisms are not fully understood. Here the authors use transcriptomic profiling in *C.* *elegans* to systematically identify the genetic pathways involved in male-induced demise, which include upregulation of a conserved ion channel that regulates fat metabolism.

## Main

Sexual interactions influence organismal health independently of reproduction in nematodes, flies and mammals^1–9^. For example, males induce weight gain and shorten lifespan in female mice, independent of fertilization^8,9^. However, the impact of sexual interactions on health is largely uncharacterized, in large part because most experiments are conducted in single-sex environments. Because of its short lifespan, *C. elegans* is an ideal model organism to systematically examine how sexual interactions affect longevity. Sexual interactions with males shorten the lifespan of the opposite sex (females or hermaphrodites) in *Caenorhabditis*^1–4^, and this phenomenon has been shown to involve components from both sexes. Indeed, males promote the premature death of hermaphrodites using male sperm and seminal fluid during mating^2^ as well as male pheromones and secreted compounds^1,10^ (especially when large numbers of males are present^11^). In hermaphrodites, several molecular and cellular pathways mediate aspects of male-induced demise, including transcription factors (for example, FOXO/DAF-16 and TFEB/HLH-30)^2,12^, chromatin regulators (for example, KDM6A/UTX-1)^1^, insulin ligands (for example, INS-11 and INS-7)^1,2,12^ and even self-sperm itself^12,13^. However, a systematic investigation of the pathways driving the negative impact of males on lifespan, and how they overlap with known longevity pathways, is still missing.

## Results

### Male-induced gene expression changes

**Fig. 1:**
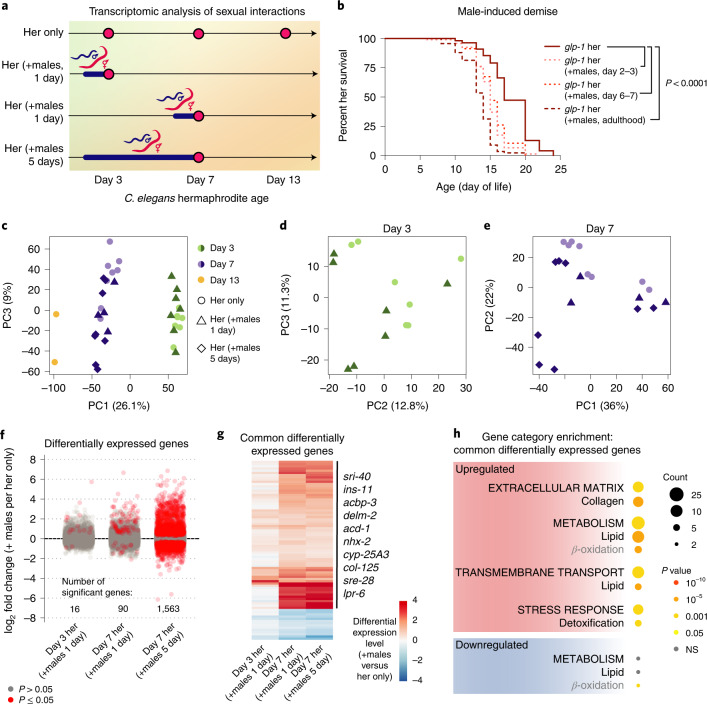
Sexual interactions induce premature death and specific transcriptional changes in *C. elegans* hermaphrodites. **a**, Scheme describing the ages of the *glp-1(e2144)* hermaphrodites (Her) used in RNA-seq experiments and length of sexual interactions with males. Pink dots, age (day of life) of hermaphrodites at sample collection times; blue line, period of time (1 day versus 5 days) with males. Three to eight biological replicates, each consisting of 75 hermaphrodites per condition. **b**, The presence of males during adulthood (either all of adulthood (dashed dark red line) or for 1 day (dotted pink and red lines)) shortens the lifespan of sterile *glp-1(e2144) C. elegans* hermaphrodites (*P* *<* 0.0001 for any condition compared with hermaphrodites only). Lifespan data plotted as Kaplan–Meier survival curves, *P* value calculated using Mantel–Cox log ranking. A total of 98–114 hermaphrodites were tested. A complete list of lifespan assay results is given in Supplementary Table 1. **c**–**e**, PCA of normalized read counts from the hermaphrodite transcriptomes after removal of male-enriched genes (Extended Data Fig. 1a,b; Methods). In **c**, data from all hermaphrodite age groups were used, and in **d** and **e**, the read counts from day 3 or day 7 only were normalized and analyzed. **f**, Male-induced hermaphrodite gene expression changes following 1 or 5 days of interaction between the sexes. After filtering the data for male-enriched genes, the log_2_(fold change) for detected genes is displayed and differentially expressed genes (*P* ≤ 0.05) in the presence of males are in red. Complete analysis results are in Supplementary Data 1. **g**, Heatmap of differentially expressed genes in hermaphrodites in the presence of males for both 1 day (day 3 or day 7 hermaphrodites) and 5 days. Data are displayed as log_2_(fold change). **h**, Selected, enriched gene categories from differentially expressed genes shared between hermaphrodites that interact with males for 1 and 5 days. Gene annotations are nested, with broadest categories in upper case letters, middle categories in lower case letters, and specific categories in gray; NS, nonsignificant. A complete gene set enrichment analysis is given in Supplementary Data 2.

To systematically assess the impact of sexual interactions with males on the opposite sex in *C.* *elegans*, we performed RNA sequencing (RNA-seq) of young (first day of adulthood or day 3 of life) and middle-aged (day 7 of life) hermaphrodites in the presence or absence of males (Fig. 1a; Methods). The males were present for a brief exposure (1 day) or a long exposure (5 consecutive days) (Fig. 1a). To eliminate possible effects of sexual interactions during development^10,14,15^ and avoid confounds from embryo transcripts, we initiated exposure to males at the onset of adulthood in sterile (*glp-1[e2144]*^16^) hermaphrodites (Fig. 1a). Male-induced demise occurs robustly and reproducibly under these conditions (Fig. 1b)^1,2^.

**Extended Data Fig. 1:**
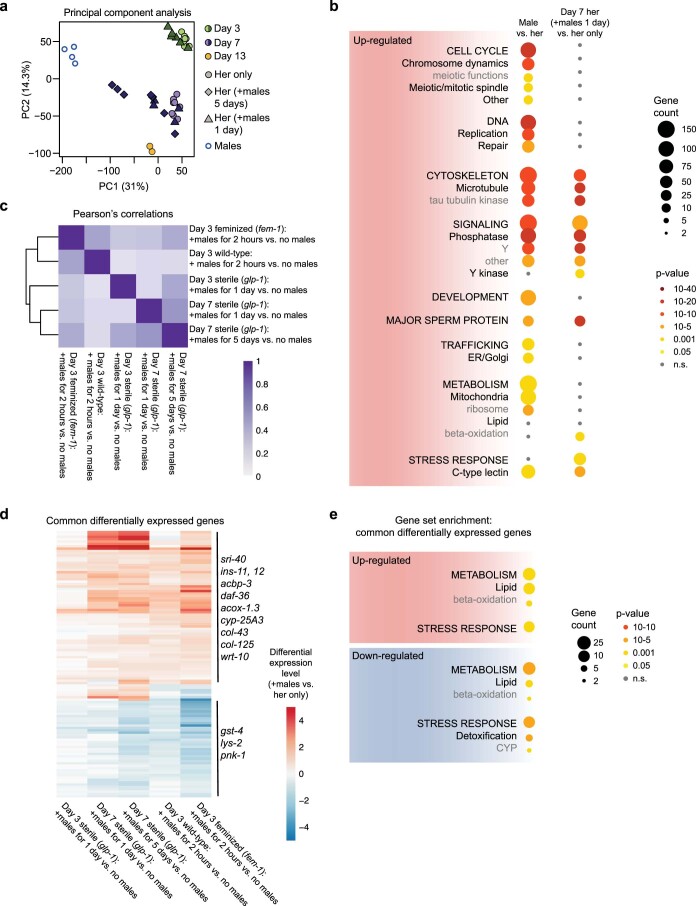
RNA-seq of *C. elegans* hermaphrodites and males. (a) Principal Component Analysis of the normalized read counts of *glp-1(e2144)* hermaphrodites and wild-type males’ transcriptomes prior to removal of male-enriched genes (see Methods). (b) Enriched gene categories in genes expressed higher in wild-type males versus *glp-1* hermaphrodites (left) and the genes expressed higher in *glp-1* hermaphrodites that experienced a sexual interaction for one day (starting day 6 of life) versus no sexual interaction (right). These two sets of differentially expressed genes share several enriched gene categories, including those linked with sperm such as the major sperm proteins, tau tubulin kinases^40^, and phosphatases/kinases^64^ suggesting that male sperm-derived transcripts are detected by whole-worm RNA-seq of mated hermaphrodites. (c) Pearson’s correlation of the DEseq2 calculated log_2_(fold change) differential expression of all shared, detected genes in wild-type, sterile *glp-1(e2144)*, and feminized *fem-1(hc17)* hermaphrodites following a sexual interaction with males versus no sexual interaction. Data are from this manuscript and Booth *et al. eLife* 2019. (d) Heatmap of genes that are differentially expressed in at least three of the five conditions. Data are displayed as log_2_(fold change). (e) Enriched gene categories in the genes that are upregulated (red) or downregulated (blue) following sexual interactions with males in at least three of the five datasets from *glp-1* sterile (this manuscript), *fem-1* feminized, and wild-type hermaphrodites^13^. In panels b and e, the number of differentially expressed genes is indicated by the size of the circle and significance of enrichment by the color of the circle (Fisher’s exact test, Bonferroni corrected). Gene annotations are nested with the broadest categories listed in all capital letters and the middle categories listed with the first letter capitalized, and the most specific categories in gray. Complete differential expression and gene set analysis results are in Supplementary Data 1 to 4.

Principal component analysis (PCA) separated the transcriptomes of the samples based on age (Fig. 1c and Extended Data Fig. 1a) and exposure to males, especially after longer exposure (Fig. 1d–e). Prolonged exposure to males resulted in a marked increase in the degree and number of gene expression changes compared with the brief exposure to males (Fig. 1f). The genes that were induced in hermaphrodites following a brief (1 day) or long (5 days) interaction with males partially overlap (for example, *ins-11*, *sri-40*, *acbp-3*) (Fig. 1g). Importantly, many of the gene expression changes that we observed in sterile, *glp-1* hermaphrodites following sexual interactions with males were also observed in wild-type, fertile hermaphrodites (Extended Data Fig. 1c–e)^13^. Genes upregulated in response to males were enriched for lipid metabolism and transport, collagens and stress response (detoxification) gene categories (Fig. 1h and Extended Data Fig. 1e). Thus, males induce striking transcriptional changes in hermaphrodites, with a greater number and magnitude of changes in response to long interactions. As both brief and prolonged exposure to males shortens hermaphrodite lifespan^1,2^ (Fig. 1b), genes that are induced in both conditions might be more functionally relevant to elicit demise and we focused on these for the remainder of the study.

### Identification of functional male-induced demise genes

**Fig. 2:**
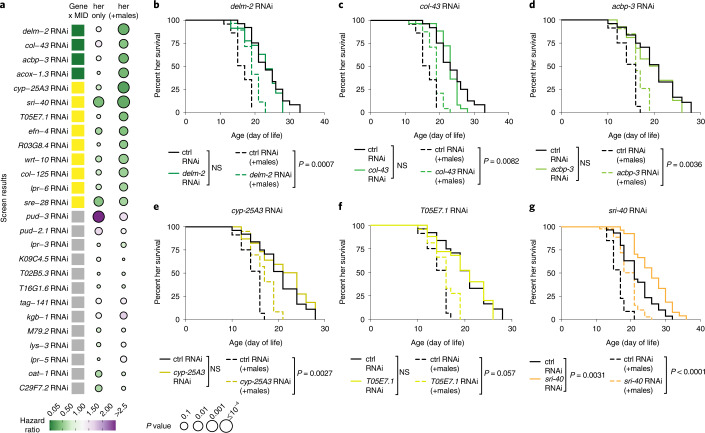
Male-induced demise is mediated by specific genes and more general health genes. **a**, Results of our RNAi-based screen for functionally important hermaphrodite genes in male-induced demise (MID). Color of squares on the left indicate significant and protective (green), significant and detrimental (purple) or NS (yellow and gray) interaction between male-induced demise and a gene knockdown using the Cox proportional hazard model (see gene classification criteria in Methods). Yellow and gray squares are distinguished based on the ability of the gene knockdown to extend lifespan in the presence of males (yellow, significant lifespan extension; gray, no significant lifespan extension). Circle colors indicate hazard ratios of specific gene knockdowns versus control, EV RNAi in hermaphrodite only conditions (left circles) or in the presence of males (right circles). Circle sizes indicate *P* values (calculated using Cox proportional hazard model). **b**–**d**, Knockdown of genes encoding the ion channel DELM-2 (**b**), collagen COL-43 (**c**), acyl-CoA binding protein ACBP-3 (**d**) or acyl-CoA oxidase ACOX-1.3 (Extended Data Fig. 2b) partially protected hermaphrodites from male-induced demise (dashed lines) but did not detectably extend lifespan in the absence of males (solid lines) (significant and protective interaction by Cox proportional hazard model). *Delm-2* knockdown can extend lifespan in the absence of males in some but not all experiments (Extended Data Fig. 2a); ctrl, control. **e**,**f**, Knockdown of the genes encoding the cytochrome P450 protein CYP-25A3 (**e**), thioesterase T05E7.1 (**f**) or other genes (Extended Data Fig. 2) extended hermaphrodite lifespan in the presence of males (no significant interaction by Cox proportional hazard model). **g**, Knockdown of serpentine receptor gene *sri-40* extended hermaphrodite lifespan in the presence (dashed lines) and absence (solid lines) of males (no significant interaction by Cox proportional hazard model). Lifespan data plotted as Kaplan–Meier survival curves and for **b**–**g**, *P* values calculated by Mantel–Cox log ranking. For the screen, 31–59 wild-type hermaphrodites were tested; the complete results of the lifespan assays can be found in Supplementary Table 1.

**Extended Data Fig. 2:**
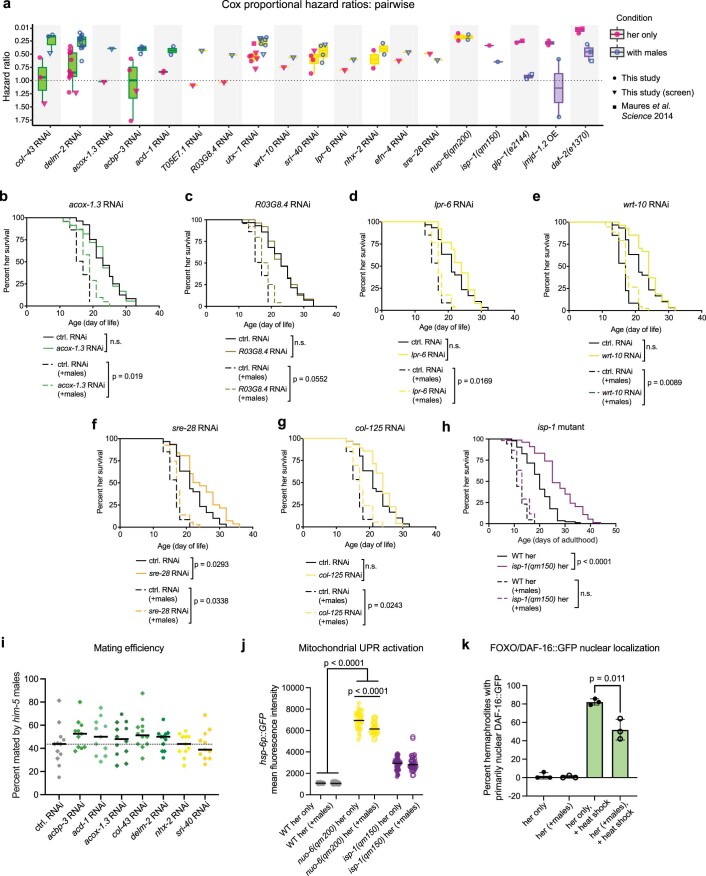
RNAi Screen Results. (a) Hazard ratios comparing the effect of RNAi knockdown or mutation to control in the absence (pink) and presence (hollow blue) of males measured by Cox Proportional Hazard Model for each independent lifespan assay. Box plot minima and maxima are the first and third quartile, respectively and center line shows the median. Whiskers extend to largest and smallest values. Some replicates of *utx-1* RNAi, *daf-2(e1370)* and *glp-1(e2144)* results were published previously^1^. (b-h) The Kaplan-Meier survival curves of the screen hits (b-g) and *isp-1(qm150)* (h). Several control RNAi data are identical because these genes were tested together in a blinded group with one control RNAi. (b-g) 32-59 wild-type hermaphrodites tested and (h) 55-113 hermaphrodites were tested. *p-*values (Mantel-Cox log ranking) shown. See Supplementary Table 1. (i) Percent of hermaphrodites that mated. There was no detectable mating efficiency difference compared to wild type. >5 plates of approximately 20 hermaphrodites per condition and replicate were tested, N=2) (j) Whole worm quantification of a mitochondrial UPR reporter (*hsp-6p::GFP)* reporter in a wild type, *nuo-6(qm200)*, or *isp-1(qm150)* background. Hermaphrodites were in the absence (filled points) or presence (hollow points) of males starting at adult day 1 and fluorescence was measured at adult day 3. In the presence of males, activation of mitochondrial UPR is blunted for *nuo-6* mutants but not WT or *isp-1* mutants (unpaired, two-tailed Mann-Whitney test for pairwise comparisons, two-way ANOVA across allele background). 29-45 hermaphrodites tested per condition. (k) Percent of hermaphrodites with primarily nuclear localization of FOXO/DAF-16. Hermaphrodites were in the presence or absence of males starting at adult day 1. Heat shock (right) and images on adult day 3. Each point for a given condition represents an independent experiment (N=3) consisting of >30 hermaphrodites per condition. Mean and standard deviation shown and *p*-values calculated by a two-tailed, unpaired t-test. Source data

To understand the functional role of these genes in the male-induced demise of hermaphrodites, we performed a targeted RNA-mediated interference (RNAi)-based screen (Fig. 2 and Extended Data Fig. 2). Using wild-type, fertile hermaphrodites, we knocked down the individual male-induced genes that we identified through RNA-seq and measured hermaphrodite lifespan in the presence and absence of males. To assess the interaction between specific genes and the presence of males for lifespan, we used the Cox proportional hazard model—a statistical method to test for association between lifespan and different parameters (Methods).

Our screen identified genes that, when knocked down, exhibited a significant and protective interaction with the presence of males by Cox proportional hazard model (green squares, Fig. 2a) and significantly extended the lifespan of hermaphrodites in the presence of males (right circles, Fig. 2a and Extended Data Fig. 2a). This category includes genes encoding the ion channel DELM-2, collagen COL-43, acyl-CoA binding protein ACBP-3 and acyl-CoA oxidase ACOX-1.3 (Fig. 2b–d and Extended Data Fig. 2b). Gene knockdowns in this category are specifically protective against male-induced demise.

We also uncovered other genes that, when knocked down, did not show a significant interaction with the presence of males by Cox proportional hazard model (yellow squares, Fig. 2a) but still significantly extended the lifespan of hermaphrodites in the presence of males (right circles, Fig. 2a and Extended Data Fig. 2a). This category includes genes encoding the serpentine receptors SRI-40 and SRE-28, and the Hedgehog-like protein WRT-10 (Fig. 2e–g and Extended Data Fig. 2c–g). Gene knockdowns in this category may be generally protective, independently of the presence or absence of males. Hence, males impinge on the health of the opposite sex in different ways—using highly specific pathways (for example, *acbp-3*, *delm-2* (displayed in green)) and more general ones (for example, *sri-40* (displayed in yellow)).

### Classical longevity mutants are susceptible to male-induced demise

**Fig. 3:**
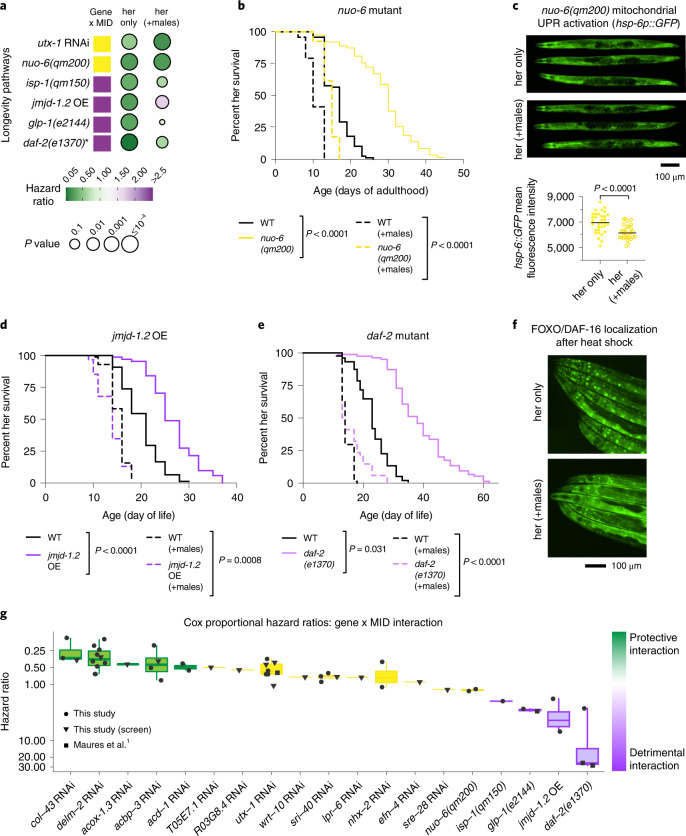
Classical longevity mutants are susceptible to male-induced demise. **a**, Comparison of classical longevity mutants, knockdown and overexpression in the presence and absence of males. Color of squares and color and size of circles as in Fig. 2a. **b**, Mutation of the mitochondria electron transport chain gene *nuo-6* extended lifespan in a hermaphrodite only setting (solid lines). In the presence of males, *nuo-6* hermaphrodites lived longer than wild type (WT), but this extension was blunted compared with the extension of lifespan in the absence of males (no significant interaction by Cox proportional hazard model). **c**, The presence of males decreases the expression of a mitochondrial UPR reporter (*hsp-6p::GFP)* under conditions that activate the mitochondrial UPR (*nuo-6* mutation). Representative images (top) and quantification (bottom, two-tailed Mann–Whitney test) are shown. The fluorescence of 29–33 hermaphrodites were measured per condition (Extended Data Fig. 2j; Source Data). **d**,**e**, Overexpression (OE) of the histone demethylase gene *jmjd-1.2* (**d**) or mutation in the insulin/IGF1 receptor gene *daf-2* (**e**) extended hermaphrodite lifespan in a hermaphrodite only environment (solid lines) but not in the presence of males (dashed lines) (significant and detrimental interaction by Cox proportional hazard model). The lifespans of 72–114 hermaphrodites were measured per condition in **b**, **d** and **e**. A complete list of lifespan data is presented in Supplementary Table 1. **f**, Representative images showing that the presence of males decreases nuclear localization of FOXO/DAF-16 following a 2-h heat shock (Extended Data Fig. 2k; Source Data). **g**, Summary of the lifespan results from our screen and additional replicates. Box plot minima and maxima are the first and third quartile, respectively, and the center line shows the median. Whiskers extend to the largest and smallest values. Hazard ratios for each lifespan assay were determined using the Cox proportional hazard model to model the interaction between male-induced demise and a gene knockdown or mutation. Inverted log_10_ scale of the *y* axis. Some replicates of the *utx-1* RNAi, *daf-2(e1370)* and *glp-1(e2144)* results were published previously^1^. All other lifespan results are from this manuscript (Supplementary Table 1). Source data

We next assessed classical long-lived mutants for the impact of the presence of males on lifespan (Fig. 3a). While a few longevity manipulations (*utx-1* RNAi^1,17,18^ and *nuo-6* mutation^19^) were also generally protective (Fig. 3a,b and Extended Data Fig. 2), several classical longevity manipulations (the insulin receptor *daf-2(e1370)* mutation^20,21^, the germline-deficient *glp-1(e2144)* mutation^22^, the mitochondrial perturbations via *isp-1(qm150)* mutation^23^ or *jmjd-1.2* overexpression^24^) exhibited a significant and detrimental interaction with the presence of males by Cox proportional hazard model (purple squares, Fig. 3a,d,e and Extended Data Fig. 2)^1,2^. Thus, several classical longevity mutants are long-lived without males but are actually more susceptible than wild type to male-induced demise, which is in sharp contrast with gene knockdowns identified in our screen. The detrimental effect of males on classical longevity mutants may be due to males repressing downstream components of longevity pathways, including FOXO/DAF-16 transcription factor nuclear localization^2^ (Fig. 3f and Extended Data Fig. 2k)^2,20–22,25^ and mitochondrial unfolded protein response (UPR) upregulation (Fig. 3c and Extended Data Fig. 2j). Thus, the context in which an individual lives (for example, with or without mates) can drastically impact the effect of a longevity intervention (Fig. 3g and Extended Data Fig. 2a).

### Genes induced by male sperm, seminal fluid and pheromones

**Fig. 4:**
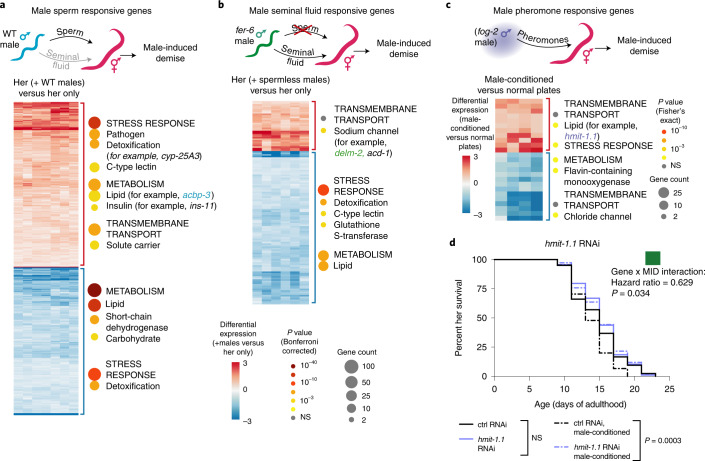
Identification of male sperm, seminal fluid and pheromone-induced genes. **a**–**c**, Heatmaps and gene set enrichment of the microarray results. Heatmap data are displayed as log_2_(fold change). Selected, enriched gene categories for the upregulated (red bracket) and downregulated (blue bracket) genes are shown to the right of each heatmap. Gene category annotations are nested with the broadest categories listed in upper case letters and categories within the broadest categories listed below, with lower case letters. Number of differentially expressed genes is indicated by the size of the circles and enrichment significance by color. Three to six biological replicates, each consisting of 100 hermaphrodites, were performed per condition. Complete results from microarrays and gene set enrichment in Supplementary Data 5–8. Male sperm-regulated hermaphrodite *glp-1[e2141]* genes identified by microarray as significantly changing in response to mating with wild-type males (that transfer sperm and seminal fluid) but not changing expression in response to mating with males that do not make sperm (*fer-6[hc6]*) (**a**). The wild-type male-mated hermaphrodite microarray results are shown. Heatmap of the putative male seminal fluid-regulated hermaphrodite genes that were identified by microarray as significantly changing both in response to mating with wild-type males that transfer sperm and seminal fluid and also with males that transfer seminal fluid but do not make sperm (*fer-6[hc6]*). The gene expression results from *fer-6* male-mated hermaphrodites are shown (**b**). Heatmap of the hermaphrodite genes that are significantly differentially expressed following exposure to male pheromones (from MCP) compared with normal plates (**c**). *P* values displayed in **c** are from the two-tailed Fisher’s exact test whereas those in **a** and **b** are the more stringent Bonferroni-corrected, two-tailed Fisher’s exact test *P* values. **d**, RNAi knockdown of the MCP-induced gene *hmit-1.1* (purple dashed line) extends the lifespan of hermaphrodites when they are cultured on MCP (*P* = 0.0003; Mantel–Cox log ranking). A significant, protective interaction between *hmit-1.1* knockdown and male-induced demise was detected by Cox proportional hazard model (*P* = 0.034). The lifespan of 107–124 hermaphrodites per condition were measured (Supplementary Table 1).

We asked whether the genes we identified are induced differently by the various male components that contribute to premature demise (male sperm, seminal fluid and pheromones^1,2,11^), as this could lead to the development of combined interventions against male-induced demise. We measured gene expression in young (day 4–5 of life) hermaphrodites following 1 day of mating with both wild-type males and sperm-less (*fer-6(hc6)*^26^) males (Fig. 4a,b and Extended Data Fig. 4a–d) (thereby distinguishing male sperm from seminal fluid) or on male-conditioned plates (MCP) for 5 days (isolating male pheromones) (Fig. 4c). Interestingly, each male component elicited a distinct transcriptional response from hermaphrodites (Extended Data Fig. 4e), consistent with the different physiological changes (for example, fat loss and body shrinking) observed in response to these male components^2,11^.

The subset of genes induced by male sperm (that is, expressed in response to mating with wild-type but not sperm-deficient males) were enriched for metabolism genes, and included genes encoding the acyl-CoA binding protein ACBP-3 and the insulin peptide involved in innate immunity INS-11 (ref. ^27^) (Fig. 4a). RNAi knockdown of the male sperm-dependent genes *acbp-3* (Fig. 2) and *ins-11* (ref. ^1^) (as well as other male sperm-induced genes) each partially protected hermaphrodites from male-induced demise (Supplementary Table 1). Thus, ACBP-3 and INS-11 are involved in premature demise and probably act in response to male sperm.

**Extended Data Fig. 3:**
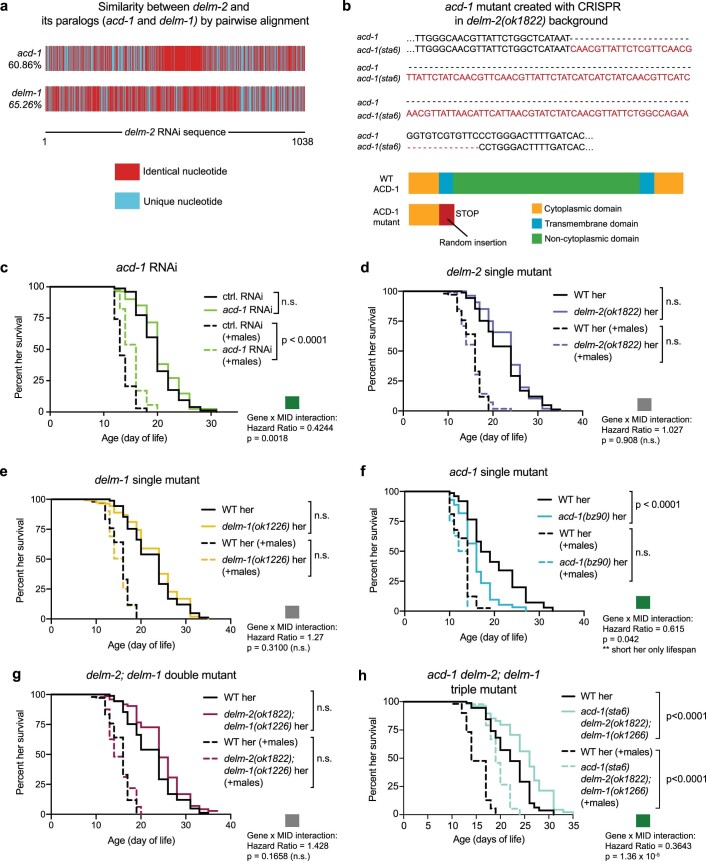
Ion channel paralogs and impact on lifespan. (a) DNA sequence alignment of the *delm-2* RNAi targeting sequence from the Ahringer RNAi library^42^ with the aligning portions of the *delm-1* and *acd-1* unspliced transcripts. (b) A schematic showing the CRISPR–Cas9 mutation we created in *acd-1* (top) and the predicted impact on the ACD-1 protein (bottom) (c) RNAi targeting *acd-1* extends lifespan of hermaphrodites in the presence of males. (d-g) Loss of function mutations in *acd-1, delm-1*, or *delm-2* alone (panels d-f) were not sufficient to extend hermaphrodite lifespan in the presence or absence of males, nor did *delm-1, delm-2* double mutation (panel g). (h) Combined mutation of *delm-2, acd-1*, and *delm-1* extended lifespan in the absence of males and, to a greater extent, in the presence of males as determined by Cox Proportional Hazard Modeling. These data suggest that ACD-1, DELM-2, and DELM-1 act redundantly. 89-138 hermaphrodites were tested in. Lifespans are plotted as Kaplan Meier survival curves, *p*-values calculated by Mantel-Cox log ranking, and 89-138 hermaphrodites were used per condition. See Supplementary Table 1 for extended statistics.

The subset of genes that were likely induced by male seminal fluid (that is, expressed both in response to mating with wild-type males as well as *fer-6(hc6)* males that cannot make sperm but still transfer seminal fluid^26^) were enriched for genes encoding sodium channel proteins such as DELM-2 and one of its paralogs, ACD-1 (refs. ^28,29^) (but not its other paralog, DELM-1) (Fig. 4b, Extended Data Fig. 3a,b and Supplementary Data 5 and 6). Similar to *delm-2* knockdown, *acd-1* knockdown specifically protected against male-induced demise (significant and protective interaction with the presence of males by Cox proportional hazard model, green squares, Extended Data Fig. 3c).

RNAi to *delm-2* or *acd-1* probably target all three paralogs (*delm-1*, *delm-2* and *acd-1*) (Extended Data Fig. 3a). To disentangle the role of each paralog in male-induce demise, we measured the lifespans of single, double and triple mutants for *delm-1*, *delm-2* and *acd-1*. While the single mutants for *delm-1*, *delm-2* or *acd-1*, and the *delm-2*;*delm-1* double mutant did not exhibit specific protection against male-induced demise (no significant interaction detected by Cox proportional hazard model, gray square, Extended Data Fig. 3d–g), the *acd-1 delm-2;delm-1* triple mutant did show a specific protection against male-induced demise (significant and protective interaction in the presence of males by Cox proportional hazard model, green square (Extended Data Fig. 3h). The *acd-1 delm-2;delm-1* triple mutant still exhibited some lifespan extension in the absence of males (though proportionally less than in the presence of males), whereas the *delm-2* RNAi knockdown did not show consistent lifespan extension in the absence of males—perhaps due to differences between knockout and knockdown (Extended Data Fig. 2a). Thus, *delm-2* and its paralogs play functional roles in male-induced demise, probably in response to male seminal fluid.

Male-secreted compounds, including pheromones, can shorten hermaphrodite lifespan in the absence of mating^1,10,11^. To identify genes involved in the response to male-secreted compounds (including pheromones), we exposed young, wild-type hermaphrodites to MCP and performed expression analysis on hermaphrodites following 5 days of exposure (Fig. 4c). Genes induced by MCP include a solute carrier/transporter gene (*hmit-1.1*). Interestingly, *hmit-1.1* RNAi (and other MCP-induced genes) protected hermaphrodites from the lifespan-shortening effect of MCP (Fig. 4d and Extended Data Fig. 5). Thus, *hmit-1.1* is a previously uncharacterized regulator of hermaphrodite demise induced by male-secreted compounds, including male pheromones.

### Effective strategies to counter male-induced demise

**Fig. 5:**
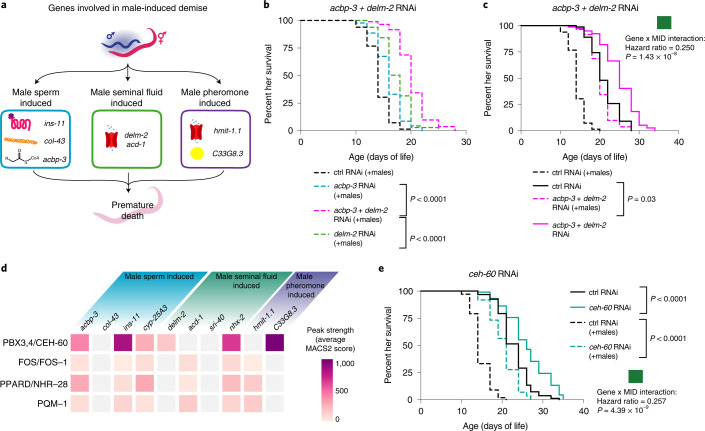
Strategies to strongly protect hermaphrodites from male-induced demise. **a**, A model for the transcriptomics and male-induced demise lifespan data showing that male sperm, seminal fluid and pheromones each induce a different set of functionally important male-induced demise gene in hermaphrodites. **b**, Reduction of *acbp-3* or *delm-2* individually by RNAi partially protected hermaphrodites from male-induced demise (*acbp-3* or *delm-2 P* < 0.0001 compared with control RNAi, Mantel–Cox log ranking). Reduction of *acbp-3* and *delm-2* simultaneously by double RNAi (dashed pink line) protected hermaphrodites from male-induced demise to a greater extent than knockdown of either *acbp-3* or *delm-2* alone (*P* < 0.0001 compared with either single gene knockdown, Mantel–Cox log ranking). **c**, Knockdown of *acbp-3* and *delm-2* simultaneously extended hermaphrodite lifespan. In the presence of males (dashed lines), loss of *acbp-3* and *delm-2* increased lifespan and resulted in a lifespan almost comparable with that of control hermaphrodites in a single-sex setting (black solid line versus dashed pink line: *P* = 0.03, Mantel–Cox log ranking). In the absence of males, loss of *acbp-3* and *delm-2* increased lifespan (*P* < 0.0001, Mantel–Cox log ranking) compared with control RNAi (solid black line). A significant, protective interaction between simultaneous *delm-2* and *acbp-3* knockdown and male-induced demise was detected by Cox proportional hazard model (*P* = 1.43 × 10^-8^). **d**, Average MACS2 score of transcription factor ChIP–seq binding peaks within ± 5 kb of the transcription start site of male-induced functional important genes. For complete transcription factor enrichment analysis, see Supplementary Data 9. **e**, Knockdown of the gene encoding the transcription factor *ceh-60* significantly extended lifespan in both the presence and absence of males (*P* < 0.0001, Mantel–Cox log ranking). Significant, protective interaction between *ceh-60* knockdown and male-induced demise by Cox proportional hazard model (*P* = 4.39 × 10^–9^). In **b**, **c** and **e**, lifespan data displayed as Kaplan–Meier survival curves. In all, 105–124 hermaphrodites were used. Complete list of lifespan assays and statistical tests are presented in Supplemental Table 1.

Different genetic pathways in the hermaphrodite mediate the demise induced by male sperm, seminal fluid and pheromones. (Fig. 4 and summarized in Fig. 5a). This observation raises the possibility that modulating these genes in combination could be more effective to counter male-induced demise. To test this, we performed lifespan assays in which we combined losses of gene function. We assessed the role of *delm-2* (induced by male seminal fluid) in combination with *acbp-3* (induced by male sperm). While reduction of each gene individually resulted in partial protection from male-induced demise, knockdown of *delm-2* (and paralogs) together with *acbp-3* resulted in additive protection against male-induced demise (Fig. 5b). Interestingly, knockdown of *delm-2* (and paralogs) and *acbp-3* in combination fully protected hermaphrodites from the lifespan-shortening impact of sexual interactions with males (Fig. 5c), and these hermaphrodites lived approximately the same lifespan as hermaphrodites in the absence of males (Fig. 5c). Thus, a combined loss of function in the hermaphrodite pathways targeted by male sperm and male seminal fluid strongly improves protection from male-induced demise.

Analysis of the regulatory regions of the genes induced by parallel male strategies revealed key transcription factor binding sites, with some of them being shared among different male-induced pathways (for example, PQM-1 (ref. ^12^), PPARD/NHR-28 and PBX3,4/CEH-60) (Fig. 5d and Extended Data Fig. 6). We tested the functional role of one of these key upstream regulators (the conserved homeobox transcription factor PBX3,4/CEH-60) and found that *ceh-60* RNAi strongly and specifically protected hermaphrodites from male-induced demise (Fig. 5e). Collectively, these results suggest that targeting the different genes induced by males in combination (using several genes or a common transcription factor) is more effective to counter male-induced demise than targeting a single gene or pathway.

### Tissue-specific regulation of male-induced demise

**Fig. 6:**
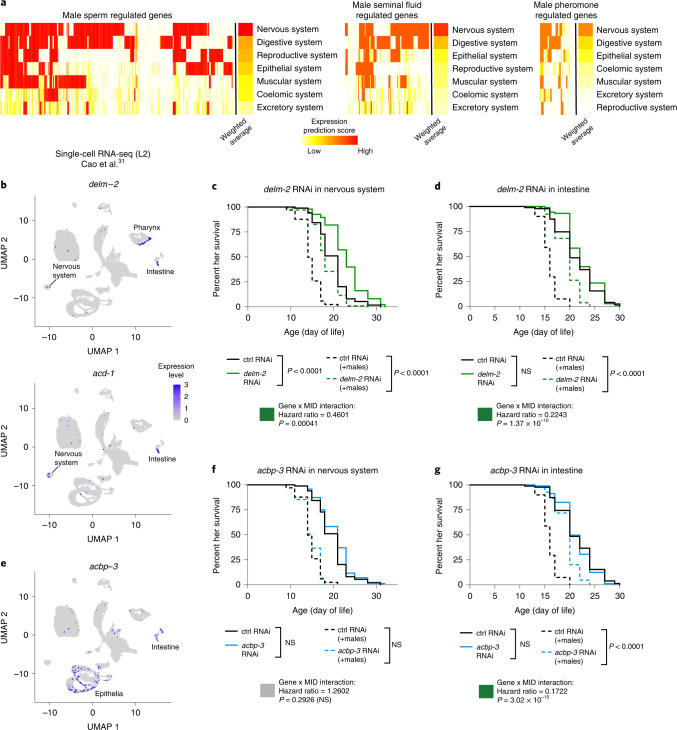
DELM-2 and ACBP-3 act in the nervous and digestive systems to regulate lifespan. **a**, Heatmaps showing the predicted tissue expression patterns^30^ of the male sperm, seminal fluid and pheromone regulated hermaphrodite genes. The weighted average score of each tissue was calculated as the mean prediction score for the male-induced genes divided by mean prediction score for all genes detected in the microarrays. **b**–**e**, UMAP plots of larval stage 2 (L2) hermaphrodite cells (**b** and **e**). Cells with detectable expression of *delm-2, acd-1* (**b**) and *acbp-3* (**e**) are shown. Previously published data^31^ was reanalyzed to generate these plots. Reduction of *delm-2* (green lines) in the nervous system (**c**) or the intestine (**d**) by RNAi is sufficient to extend lifespan of hermaphrodites in the presence of males (dashed lines, *P* < 0.0001) and conferred specific protection against male-induced demise as shown by modeling the interaction between male-induced demise and tissue-specific *delm-2* RNAi using Cox proportional hazard modeling (*P* = 0.00041 for nervous system specific RNAi and *P* = 1.37 × 10^–10^ for intestine-specific RNAi). *delm-2* RNAi occasionally results in small extension of lifespan in the absence of males (Extended Data Fig. 2a and Supplementary Table 1). **f**, Knockdown of *acbp-3* (blue lines) by RNAi specifically in the nervous system had no detectable impact on hermaphrodite lifespan in the presence (dashed lines) or absence (solid lines) of males. **g**, In contrast, RNAi knockdown of *acbp-3* specifically in the intestine extended lifespan of hermaphrodites when in the presence of males (dashed lines, *P* < 0.0001) but not in the absence of males (*P* = 3.02 × 10^–15^ using Cox proportional hazard modeling of the interaction between male-induced demise and intestine-specific *acbp-3* RNAi). Lifespan data are plotted as Kaplan–Meier survival curves and *P* values calculated by Mantel–Cox log ranking. A total of 98–111 hermaphrodites were used per condition. See Supplementary Table 1 for a complete list of all lifespan data.

We asked whether the genes induced by males act in specific cells and tissues to mediate male-induced demise. Using a prediction tool based on tissue-specific expression^30^, we found that the genes differentially expressed by exposure to male sperm, seminal fluid and pheromones were all enriched in the nervous system and the digestive system (for example, intestine) (Fig. 6a). Genes involved in the response to sperm and seminal fluid were also enriched in the reproductive and epithelial system (Fig. 6a). We focused on two of the top genes mediating male-induced demise: the seminal fluid-responsive gene *delm-2* and the sperm-responsive gene *acbp-3*. To analyze their expression in different cells, we used publicly available single-cell RNA-seq data (L2 hermaphrodites)^31^ (Fig. 6b,e). This analysis revealed that *delm-2* and paralog *acd-1* were each expressed in intestinal and nervous system cells, and that *delm-2* was expressed in pharyngeal epithelial cells (Fig. 6b). *acbp-3* was expressed in intestinal and epithelial cells (Fig. 6e). Together, this analysis suggests a multi-tissue response, with contribution from both the nervous and digestive systems.

We next determined if DELM-2 (and paralogs) and ACBP-3 could functionally mediate male-induced demise by acting in these tissues. Using tissue-specific RNAi, we showed that reduction of *delm-2* and paralogs in either the nervous system or the intestine was sufficient to protect against male-induced demise (Fig. 6c,d). In contrast, reduction of *acbp-3* in the intestine, but not in the nervous system, protected against male-induced demise (Fig. 6f,g). Hence, DELM-2 and paralogs function in at least two tissues—the nervous system and the intestine—to mediate male-induced demise.

### Regulation of fat metabolism by the ion channel DELM-2

**Fig. 7:**
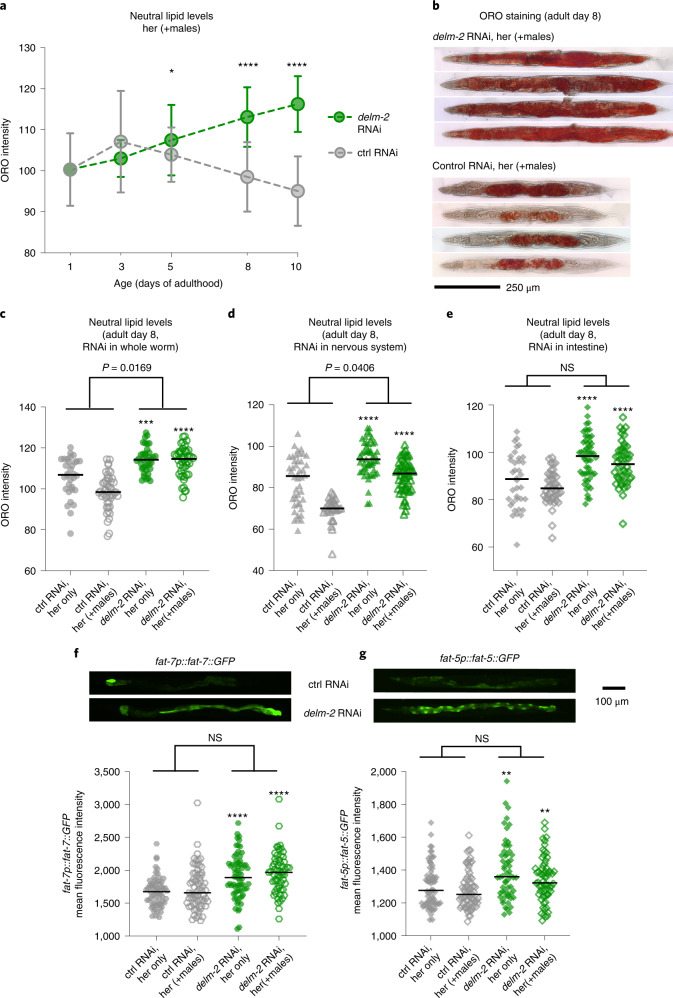
DELM-2 modulates fat metabolism by acting in the nervous system and intestine. **a**, Timecourse of whole-worm neutral lipid levels (measured by ORO staining) in hermaphrodites in the presence of males starting at adult day 1 and cultured on control (gray) or *delm-2* (green) RNAi bacteria. Mean and s.d. are shown. *P* values calculated by two-tailed, unpaired Mann–Whitney test (*delm-2* RNAi versus control on day 5 *P* = 0.0336 and on days 8 and 10 *P* < 0.0001). **b**, Representative images of ORO-stained adult day 8 hermaphrodites in the presence of males starting at adult day 1 and cultured on *delm-2* RNAi (top panel) or control RNAi bacteria (bottom panel). Images are at the same scale for all conditions. **c**–**e**, Neutral lipid levels of adult day 8 hermaphrodites in the absence (filled points) or presence (hollow points) of males as measured by whole-worm ORO intensity. Knockdown of *delm-2* by RNAi (green points) in the whole worm ((**c**) her only, *P* = 0.0002; +males, *P* < 0.0001), only in the nervous system ((**d**) her only, *P* < 0.0001; +males, *P* < 0.0001) or only in the intestine ((**e**) her only, *P* < 0.0001; +males, *P* < 0.0001) significantly increased neutral lipid level in both the presence and absence of males (asterisks, comparing *delm-2* versus control RNAi, unpaired, two-tailed Mann–Whitney test). *delm-2* RNAi in the whole worm (**c**) or only the nervous system (**d**) also decreased male-induced neutral lipid loss (*P* = 0.0169 and *P* = 0.0406, respectively, two-way ANOVA). **f**,**g**, Knockdown of *delm-2* increased expression of the desaturases (**f**) FAT-7 and (**g**) FAT-5 in adult day 3 hermaphrodites in the absences (filled points) and presence (hollow points) of males (asterisks, comparing *delm-2* versus control RNAi, unpaired, two-tailed Mann–Whitney test: (**f**) her only, *P* < 0.0001; +males, *P* < 0.0001; (**g**) her only, *P* = 0.0013; +males, *P* = 0.0022). The presence of males did not detectably impact expression of these desaturases (two-way ANOVA). FAT-7 and FAT-5 levels in the whole worm were quantified using protein fusion reporters. Representative images shown in the top panel. A total of 20–62 hermaphrodites were measured per condition and replicate in **a** and **c**–**g**. Asterisks indicate *P* values from the Mann–Whitney test (* ≤0.05, ** ≤0.01, *** ≤0.001, **** ≤0.0001). Complete results of the ORO and fluorescence reporter quantification in Source Data. Source data

How might DELM-2 regulate male-induced demise? Males induce changes in lipid metabolism in the opposite sex in several species^2,6,32,33^ and promote depletion of neutral lipids (triglycerides) in *C. elegans*^2,32^. We therefore determined whether DELM-2 could regulate lipid metabolism in the absence and presence of males. To measure neutral lipids, we performed Oil Red O (ORO) staining on fixed hermaphrodites. We found that *delm-2* RNAi or mutation of *delm-2* and its paralogs significantly prevented the depletion of neutral lipids induced by males, especially at middle age (Fig. 7a–c; Source Data). Reduction of *delm-2* (and paralogs) either in the intestine or the nervous system increased neutral lipids both in the presence and absence of males, and, interestingly, reduction of *delm-2* (and paralogs) in the nervous system prevented the depletion of neutral lipids that is normally induced by males (Fig. 7d,e). Knockdown of *delm-2* (and paralogs) also upregulated expression of enzymes (for example, FAT-5 and FAT-7) involved in lipid metabolism, notably the production of mono-unsaturated fatty acids (MUFAs) (Fig. 7f,g). These results are consistent with the possibility that loss of the ion channel DELM-2 and paralogs could protect hermaphrodites from male-induced demise by regulating MUFA synthesis and preventing fat loss induced by males. These observations also suggest a multi-tissue site of action for DELM-2 and paralogs in regulating fat metabolism.

## Discussion

Here we have identified genes that are not only regulated by but are also functionally important for the response of hermaphrodites to sexual interactions (male-induced demise). Our analysis uncovers specific regulators of male-induced demise (for example, ACPB-3 and DELM-2 and paralogs). It also identifies regulators whose reduction extends lifespan both in the absence and presence of males (for example, SRI-40) and that have been missed by previous genetic screens. In contrast, several classical longevity mutants are, in fact, more susceptible to the presence of males than wild type, with males inhibiting components of classical longevity pathways. Thus, several classical long-lived mutants may have an Achilles’ heel: their susceptibility to the opposite sex. Our study reveals that targeting genes in a mixed- versus a single-sex environment can have different outcomes on lifespan, which has important implications for health. Sexual interactions may therefore represent a particularly potent biological force distinct from other types of stresses.

Why have hermaphrodites not evolved to turn the genes induced by males off? One possibility could be that these genes provide some evolutionary benefit to the hermaphrodites that outweighs their cost. Alternatively, negative selection may be weak on these genes because *C. elegans* males are not normally abundant and because hermaphrodites succumb to male-induced demise after reproducing. A third possibility is that the induction of these genes by males provides a benefit to the next generation that might offset the costs of males to maternal health.

By investigating these genes through different ‘lenses’ we have found that they fall into different categories both in terms of the male signal to which they respond (male sperm, seminal fluid or pheromones) and the specificity of their regulation of lifespan (specific to male-induced demise versus broad longevity regulation). Strikingly, many classical longevity mutants are more susceptible than wild-type animals are to male-induced demise, possibly through repression of their downstream pro-longevity effectors (for example, DAF-16/FOXO^2^). Future studies on the effects of sexual interactions on classic longevity pathways will be important to further elucidate the mechanism of action. Many of the genes identified in this study are conserved in mammals and could play a role in lifespan and health in humans. The placement of male-induced demise genes in different pathways allowed us to predict which combination of strategies could lead to greater protection from male-induced demise. Indeed, knockdown of *delm-2* (and paralogs) and *acbp-3* in combination (or knockdown of a gene encoding a potential common regulator, the transcription factor CEH-60) strongly extends the lifespan of hermaphrodites in the presence of males to the lifespan of hermaphrodites in the absence of males. This is important because it could help identify additional combinations of strategies that more robustly extend lifespan.

Finally, we find that DELM-2, a conserved ion channel, is specifically involved in male-induced demise and regulates lipid metabolism. The ability of DELM-2 and paralogs to impact the enzymes involved in MUFA production is consistent with the observation that MUFAs themselves partially protect hermaphrodites from male-induced demise^32^. DELM-2 and paralogs act in the nervous system and intestine to regulate lifespan and lipid metabolism in response to sexual signals received in the reproductive system (male seminal fluid). This observation suggests that communication between tissues is important for male-induced demise. The specific molecules involved in the tissue-to-tissue communication are unknown, but intriguingly, mammalian orthologs of *delm-2* (SCNN1G and ASIC1,2,3) are involved in sensing specific lipids^34,35^. Overall, our study reveals that longevity is highly dependent on the sexual environment (single sex versus mixed sex) and that sexual interactions trigger an elaborate network of functional regulation, which could extend to other species and be used to counter aging and age-related diseases.

## Methods

### Worm strains and maintenance

**Table:** 

*C. elegans* strain	Genotype	Source
N2	Wild type	M.-W. Tan & CGC^a^
CF1903	*glp-1(e2144) III*	CGC
CB4037	*glp-1(e2141) III*	CGC
CB1467	*him-5(e1467) V*	CGC
BA6	*fer-6(hc6) I*	CGC
CB4108	*fog-2(q71) V*	CGC
RB1523	*delm-2(ok1822) I*	CGC and CDMC^b^
RB1177	*delm-1(ok1226) IV*	CGC and CDMC
BLC100	*delm-1(ok1226) IV; delm-2(ok1822) I*	L. Bianchi
ZB90	*acd-1(bz90) I*	L. Bianchi
CB1611	*mec-4(e1611) X*	M. Goodman
ABR212	*acd-1(sta6) delm-2(ok1822) I*	This study
ABR225	*acd-1(sta6) delm-2(ok1822) I ; delm-1(ok1226) IV*	This study
MQ1333	*nuo-6(qm200) I*	CGC
MQ887	*isp-1(qm150) IV*	CGC
CB4876	*clk-1(e2519) III*	CGC
CB1370	*daf-2(e1370) III*	CGC
AGD1505	*uthIs404[myo-2p::tdtomato, sur-5p::jmjd-1.2a::3’UTR unc-54]*	A. Dillin
SJ4100	*zcIs13 [hsp-6p::GFP + lin-15(+)]*	CGC
ABR213	*nuo-6(qm200) I; zcIs13 [hsp-6p::GFP + lin-15(+)]*	This study
ABR214	*isp-1(qm150) IV; zcIs13 [hsp-6p::GFP + lin-15(+)]*	This study
TJ356	*zIs356 [daf-16p::daf-16a/b::GFP +rol-6(su1006)]*	CGC
TU3401	*sid-1(pk3321) V; uIs69 [pCFJ90(myo-2p::mCherry) + unc-119::sid-1]*	CGC
VP303	*rde-1(ne219) V; kbIs7 [nhx-2p::rde-1 + rol-6(su1006)]*	CGC
IG1839	*Rde-1(ne300) V; frSi17 [mtl- 2p::rde-1 3’UTR] II. frIs7 [nlp-29p::GFP + col-12p::DsRed] IV*	CGC
BX113	*lin15B, A(n765) X; waEx15 [fat-7::GFP + lin15(+)]*	CGC
BX150	*lin15B, A(n765) X; waEx18 [fat-5::GFP + lin15(+)]*	CGC

^a^CGC, *Caenorhabditis* Genetics Center

^b^CDMC, *C. elegans* Deletion Mutant Consortium^36^

All *C. elegans* wild-type and mutant strains used in this study are listed above. All strains were maintained on Nematode Growth Media (NGM) plates with 50 μg ml^–1^ streptomycin (Gibco) and a lawn of OP50-1 bacteria (a gift from M.-W. Tan) from stationary phase cultures. Nematodes were grown at 20 °C, except temperature-sensitive mutants (*glp-1(e2144)* and *(e2141)*), which were maintained at 15 °C (permissive temperature). When temperature-sensitive mutants were used for assays, they were grown at the restrictive temperature (25 °C). The genotype of strains was verified by genotyping PCR and Sanger sequencing and the strains were backcrossed three times into our laboratory’s N2 strain (in addition to the backcrossing that was performed when the mutants were initially isolated).

### RNA-sequencing

To better understand how males induce premature hermaphrodite demise, we characterized the transcriptomes of sterile *glp-1(e2144)* hermaphrodites that have a shortened lifespan after interacting with wild-type males for 1 day or 5 days to those that never interacted with males.

Individuals were age-synchronized using a brief, 3- to 4-h egg lay (Lifespan assays) on 10-cm NGM plates seeded with OP50-1 bacteria and grown at 25 °C during development and adulthood. The day of the egg lay is considered day 0 of life. For the longer (5 day) exposure to males, 75 *glp-1(e2144)* hermaphrodites were placed onto a 10-cm NGM plates seeded with OP50-1 bacteria and with 75 young (day 3–5 of life) wild-type males starting on day 2 of life (young adults) until day 7 of life. At day 5 of life, the hermaphrodites were moved to fresh plates and the males were replaced with new, young wild-type males. For samples in which hermaphrodites interacted with males for a single day, 75 *glp-1(e2144)* hermaphrodites were placed onto a 10-cm NGM plate seeded with OP50-1 bacteria and 75 young (day 3–5 of life) wild-type males were added starting on either day 2 of life (young adults) or on day 6 of life. Hermaphrodites that interacted with males starting on day 6 of life were maintained on 10-cm NGM plates with OP50-1 at a density of 150 hermaphrodites starting from day 2 of life. At the same time, hermaphrodites from the same cohort of age-synchronized *glp-1(e2144)* individuals were also placed on fresh plates without males (150 hermaphrodites per 10-cm plate to maintain a similar density). After 1 or 5 days, 75 hermaphrodites from each condition (individuals that either interacted with males or never interacted with males) were collected by removing either 75 hermaphrodites (for the ‘no males’ condition) or the 75 males from the plates. For each sample, these remaining 75 hermaphrodites were immediately washed three times with ice-cold M9 buffer (22 mM KH_2_PO_4_, 42 mM Na_2_PO_4_, 86 mM NaCl and 1 mM MgSO_4_) and the worm pellets were flash frozen in liquid nitrogen. In parallel with the RNA-seq sample collection, *glp-1(e2144)* hermaphrodites and wild-type males form the same batches of age-synchronization as their corresponding RNA-seq samples were used to measure hermaphrodite lifespan (Lifespan assays; Supplementary Table 1).

To determine the contribution of male transcripts to the *glp-1(e2144)* samples, we also isolated and prepared for RNA-seq the population of wild-type males that were on the RNA-seq replicates E–H plates with the day 6–7 of life, single-day sexual interaction condition. These young male worms (not synchronized) were washed with ice-cold M9 buffer and flash frozen as described above.

RNA was extracted from the flash frozen worm pellets (approximately 75 whole worms per sample) with 500 μl Trizol and 200 μl chloroform followed by 250 μl phenol and 200 μl chloroform extractions and, finally, an isopropanol precipitation. Remaining DNA was degraded with DNaseI (Promega) and the RNA cleaned with a sodium acetate and ethanol precipitation. RNA quality was measured using Nanodrop spectrophotometry and the Agilent BioAnalyzer Total RNA Nano chip and kit. mRNA enriched cDNA was prepared using 10 ng total RNA (quantified by Nanodrop spectrophotometry) and the Takara SMART-seq v.4 Ultra Low Input RNA kit, with eight rounds of amplification. Paired-end libraries were made using the Nextera XT DNA library prep kit (Illumina) with 1 ng cDNA (quantified using the Qubit dsDNA High Sensitivity reagents, Invitrogen) and barcoded using the Nextera XT Index Kit v.2 (Illumina). Libraries were purified with 30 μl AMPure XP beads (Beckman Coulter) as directed in the Nextera XT kit. Library quality and quantity were assessed using the Agilent Bioanalyzer High Sensitivity DNA Assay. The libraries for biological replicates A–D were prepared together and pooled and sequenced on a single Illumina NextSeq run and the libraries for biological replicates E–H were prepared together and pooled and sequenced on two Illumina NextSeq runs. Paired-end, 75 base-pair sequencing was performed. All RNA-seq reads are publicly available through NCBI Sequence Read Archive (BioProject PRJNA642294).

### RNA-seq analysis

RNA-seq reads were aligned to the WBcel235 genome and gene read counts were calculated using STAR (v.2.5.4a)^37^. Low-coverage genes that had less than one read count per million mapped reads in fewer than three samples were filtered out. After filtering of low-coverage genes, 16,706 genes remained (before removal of male-enriched genes) with read coverage per sample ranging from 3,650,980 for the lowest quantile and 31,137,429 for the highest quantile. Data were normalized with a variance-stabilizing transformation (DESeq2 v.1.10.1)^38^ before PCA in R (v.3.2.4 and Biobase v.2.30.0 and v.2.42.0 (ref. ^39^)). PCA was carried out using the R method (prcomp). Differential expression was calculated using DESeq2 (v.1.10.1)^38^. The results from DESeq2 can be found in Supplementary Data 1 and 3. Heatmaps were generated in R using pheatmap (v.1.0.12) from normalized read counts (variance-stabilizing transformation). Dot plots were created with ggplot2 (v.3.3.0) and Venn diagrams with vennerable (v.3.1.0.9000).

Hermaphrodites that have interacted with males receive male sperm as a result of mating and male-sperm-derived transcripts were detected in our data (Extended Data Fig. 1). To focus on the effect of sexual interactions of the hermaphrodites, we developed a list of male-enriched genes that were excluded from future analysis. Briefly, we used DEseq2 to calculate differential expression between the wild-type males and the *glp-1(e2144)* hermaphrodites that never experienced a sexual interaction (day 3 and 7 of life were combined for this analysis). Genes that were expressed more highly in wild-type males (log_2_(fold change) > 0, adjusted *P* value ≤ 0.05) were excluded from the datasets. A total of 5,355 genes met this threshold and were called as male-enriched. This filtered dataset was then carried through our standard RNA-seq pipeline. Both the nonfiltered datasets and the datasets in which the male-enriched genes were removed from the analysis are available as supplemental datasets and their analyses are included in Supplementary Data 1 and 3.

All code is publicly available online (https://github.com/brunetlab/Booth-et-al.-2022).

### Gene set enrichment analysis

WormCat^40^ was used to determine the gene set enrichment for the RNA-seq and microarray results. Genes were identified as significant by significance analysis of microarrays (SAM) and DESeq2. For RNA-seq, genes were called as differentially expressed if the adjusted *P* value was less than 0.05. Significantly up- and downregulated genes were input to http://www.wormcat.com/ using default settings. For gene sets with a very small number of genes (those up- and downregulated on MCP), we did not observe significant enrichment using the multiple-hypothesis corrected *P* values and instead present the Fisher’s exact test for these gene sets. Complete lists of all WormCat gene set enrichment results, including both the Fisher’s exact test and Bonferroni-corrected *P* values are presented in Supplementary Data 2, 4 and 8.

### Lifespan assays

*C. elegans* hermaphrodites used for lifespan assays were age-synchronized with a short (3–4 h) egg lay using young (day 3–5 of life), well-fed, adult parents. All worms were grown on NGM plates with streptomycin (50 μg ml^–1^) and seeded with OP50-1 bacteria unless RNAi knockdown was performed.

For each assay, worms were scored as dead or alive and transferred to new plates daily during the reproductive period and then every other day. Worms were scored as dead if they did not respond to gentle, repeated prodding with a wire pick (90% Pt, 10% Ir) along different points of their body. Worms were scored as censored if they crawled off the media or died due to bagging (internal hatching) or vulval rupture. Data from these censored worms were included up until the point of censorship (see Supplementary Table 1 for all data). While blinding was not possible for comparing hermaphrodite in the absence or presence of males, blinding was done for comparing genotypes and/or RNAi knockdown.

For conditions in which the effect of sexual interactions was assessed, we used one of three methods, as indicated. For the long-term exposure method (described previously^1,2^), young males (day 1 to 2 of adulthood) were added to the hermaphrodites at the onset of adulthood. For lifespan experiments in which the hermaphrodites were exposed to males for their entire adulthood^1^, males were added in a 1:1 ratio with hermaphrodites and the number of males remained fixed, even as hermaphrodites began to die or censored. Male worms were replaced every other day at the time the hermaphrodites were transferred to new plates. Male stocks were set up approximately every other day for the entirety of the lifespan assay. For the lifespan experiments in which hermaphrodites were exposed to males for only 1 day^2^, young males were added in a 2:1 male to hermaphrodite ratio. Following 24 h of exposure, hermaphrodites were moved to new plates and did not encounter a male again throughout their lifespan. For the male-conditioned media lifespan assays, hermaphrodites were transferred onto MCP from late L4 stage and stayed on MCP for the remainder of their lives. MCP were prepared throughout the course of the lifespan assays: 30 day 1 of life males (*fog-2(q71)*, essentially wild type) were transferred onto each plate (35-mm NGM plates); 2 days later, the males were removed and hermaphrodites for lifespan assays were immediately transferred onto these MCP.

Synchronized worms (hermaphrodites, feminized individuals, and so on) were randomly assigned to the ‘no males’ or ‘+ males’ conditions by picking them onto fresh plates in an alternating manner to avoid selection bias. Similarly, the males used for mating with individuals of different genotypes or RNAi treatments were from the same sets of males in each assay and were allocated randomly in an alternating manner. For each single biological replicate, approximately 35 individuals were placed on each of three to six plates (each plate represents a technical replicate). The number of individuals per plate and number of technical replicates were chosen based on field standards^41^.

For sterile mutants, slight modifications were made to the methods. The sterile *glp-1(e2144)* mutant and wild-type control parents were used for an egg lay at the permissive temperature (15 °C). Following the egg lay, the individuals used for the assay were kept at 25 °C for the remainder of the assay.

The number of animals (*n*) used for each assay and the number of independent biological replicates (*N*) can be found in Supplementary Table 1. Lifespan data were plotted as Kaplan–Meier survival curves and pairwise statistical analyses were performed using the logrank (Mantel–Cox) test in Prism v.8 and the Cox proportional hazard model in R (v.3.5.1) to model the effect of a gene knockdown or mutation in either the presence or absence of males. Figure panels summarizing lifespan assay results (for example, box plots) were made in R using ggplot2 (v.3.3.0). The R code used for Cox proportional hazard modeling and for graphing data is available at https://github.com/brunetlab/Booth-et-al.-2022.

### RNAi knockdown

To knock down expression of specific genes, we fed worms HT115 (*Escherichia coli*) bacteria expressing double-strand RNA targeting a specific gene. For whole-worm RNAi knockdown, wild-type (N2) hermaphrodites were used. For tissue-specific RNAi knockdown, TU3401 (neuron-specific), VP303 (intestine-specific) or IG1839 (intestine-specific) hermaphrodites were used. Worms were cultured on NGM containing ampicillin (100 μg ml^–1^, Sigma) and isopropyl-β-d-thiogalactoside (IPTG) (0.4 mM, Invitrogen). During development, worms were fed HT115 bacteria (grown to stationary phase, RNAi expression induced for 2–4 h with 0.4 mM IPTG, and the bacteria concentrated to 20×) carrying control empty vector (EV). Upon adulthood (day 3 of life), worms were placed onto plates with HT115 bacteria (grown to stationary phase, RNAi expression induced for 2–4 h with 0.4 mM IPTG, and the bacteria concentrated to 20x) carrying the appropriate RNAi clone. RNAi clones in HT115 *E. coli* were isolated from the Ahringer RNAi library^42^ (a gift from A. Fire) or, if unavailable, from the Vidal RNAi library^43^ (Dharmacon). The inserts of the plasmids encoding the RNAi clones used in this study were sequenced to verify their identity. For all lifespan assays, the identity of the RNAi clone was blinded until the lifespan assay was completed.

**Extended Data Fig. 4:**
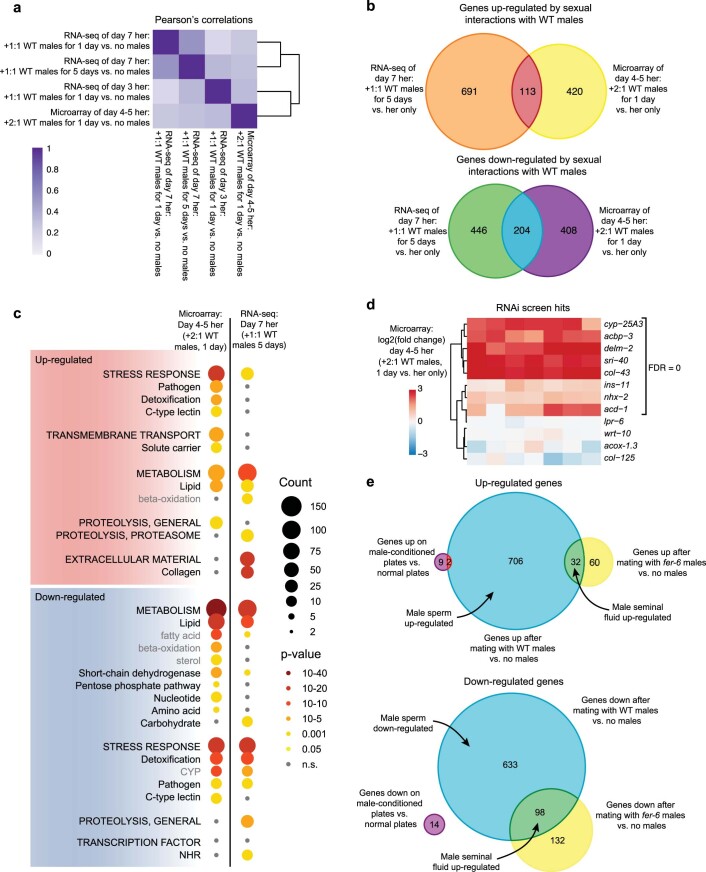
Comparison of RNA-seq and microarray results. (a) Pearson’s correlation scores comparing the wild-type male-induced fold-change in hermaphrodite gene expression detected in the *glp-1* hermaphrodite RNA-seq and microarray. (b) Venn diagrams of the wild-type male-induced differentially expressed hermaphrodite genes detected by microarray and RNA-seq. The number of overlapping differentially expressed genes is higher than expected by chance (upregulated: *p*=6.15x10^-21^, downregulated: *p*=1.69x10^-93^, hypergeometric test). (c) Enriched gene categories in the hermaphrodite genes that are upregulated (red) and downregulated (blue) by wild-type males in the microarray and the RNA-seq results. The number of differentially expressed genes in each gene set is indicated by the size of the circle and the significance of the enrichment by the color of the circle. Gene annotations are nested with the broadest categories listed in all capital letters and the middle categories listed with the first letter capitalized, and the most specific categories in gray. (d) A heatmap of the microarray results from *glp-1* hermaphrodites mated with WT males for one day. The genes that we identified as functionally important for male-induced demise (Fig. 1i and Extended Data Fig. 2) are shown, including *ins-11*^1^. The majority of genes that were differentially expressed in our RNA-seq and identified as functionally important in our screen were also differentially expressed in the microarray experiment. The four genes that were not significantly upregulated under the different experimental conditions used for the microarray were not investigated further. *sre-28* was not detected in the microarray. (e) Venn diagrams of the significantly up- and down-regulated hermaphrodite genes detected by microarray following one day of interacting (mating) with WT males or *fer-6(hc6)* sperm-less males (blue and yellow, respectively) or five days on male-conditioned plates (that is male-secreted compounds and pheromones, purple). Data from the RNA-seq and microarrays can be found in Supplementary Data 1, 2 and 5 to 8.

**Extended Data Fig. 5:**
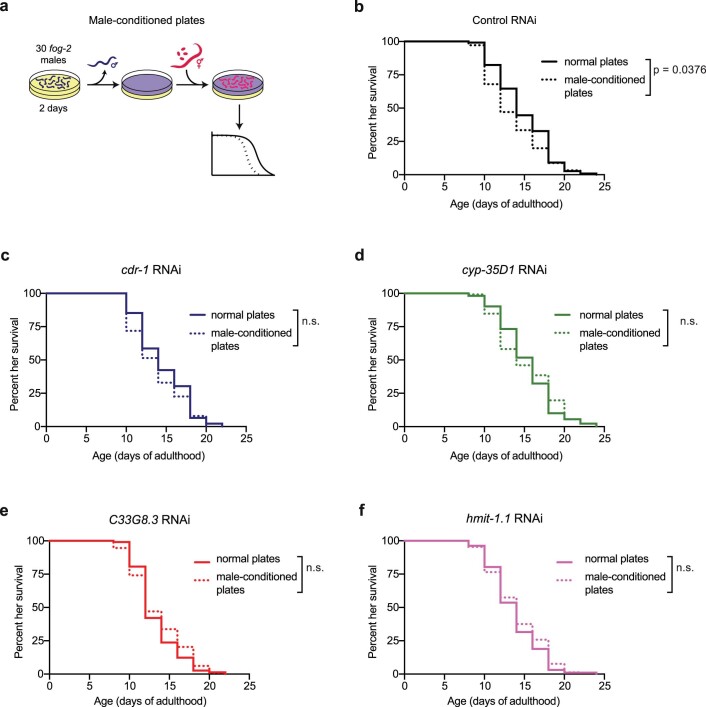
Male-conditioned plate screen results. (a) A scheme describing the experimental set up for the male-conditioned plate lifespan assays. For complete details, see Methods. (b-f) The lifespans of hermaphrodites fed control empty vector RNAi (b) or RNAi against the male-conditioned plate induced genes *cdr-1* (c), *cyp-35D1* (d), *C33G8.3* (e), or *hmit-1.1* (f). Hermaphrodite worms were either exposed to media conditioned by 30 *fog-2(q71)* males for 2 days (removed prior to placing the hermaphrodites on the plate) (dotted lines) or were placed on normal plates (solid lines). Results are plotted as Kaplan-Meier survival curves and *p*-values calculated using Mantel-Cox log ranking. Percent change in median lifespan compared to normal plates is shown in each panel. 118-132 hermaphrodites were tested in each condition. See Supplementary Table 1 for a complete list of all lifespan assay results.

**Extended Data Fig. 6:**
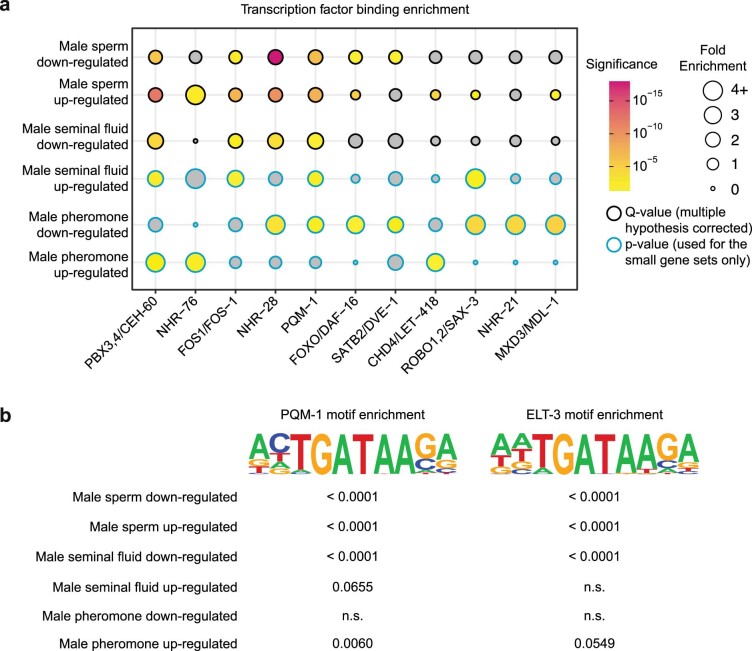
Transcription factor binding enrichment. (a) The enrichments of selected transcription factor binding peaks within 2kb +/- the transcription start site of the male sperm, seminal fluid, and pheromone downregulated and upregulated genes are shown. Fold enrichment at the gene set of interest compared to genome-wide is indicated by the size of each circle and Q-value (Benjamini-Hochberg false discovery corrected two-tailed Fisher’s exact test *p*-value) or *p*-value (two-tailed Fisher’s exact test) by the color of each circle. A complete list of the transcription factor binding enrichment is presented in Supplementary Data 9. (b) Enrichment of ‘known’ transcription factor binding motifs within 300bp +/- the transcription start site of the differentially expressed genes. The Bonferroni-corrected, one-tailed Fisher’s exact test *p-*values for the motif enrichment for each gene set is shown.

We note that the RNAi construct that targets *delm-2* shares high sequence similarity to two *delm-2* paralogs: *delm-1* and *acd-1* (Extended Data Fig. 3). Interestingly, *acd-1* expression was also induced by males (Fig. 1g and Extended Data Fig. 4d) and loss of *acd-1* by RNAi knockdown partially protected hermaphrodites from male-induced demise (Fig. 1m and Supplementary Table 1). However, single mutations in these genes are not sufficient to protect hermaphrodites from male-induced demise (Extended Data Fig. 3), suggesting that these channel genes may act redundantly or that the RNAi knockdown results in a greater loss of function either of a single gene or a combination of *delm-2, delm-1* and *acd-1*. To test this possibility, we generated an *acd-1* and *delm-2* double mutant and an *acd-1, delm-2*, and *delm-1* triple mutant (see below, CRISPR–Cas9 knockout of *acd-1*).

To perform double RNAi, we combined equal amounts of bacteria expressing *delm-2* targeting dsRNA and *acbp-3* targeting dsRNA. This was compared with control RNAi bacteria (with EV) and to single RNAi knockdown. The single RNAi knockdown for these experiments were diluted 50% using control (EV) RNAi expressing bacteria. We note that RNAi knockdown does not result in complete loss of function (that is, it is not a null). Therefore, a caveat to the interpretation of double RNAi results is that effects on lifespan may be due to intensifying the loss of function in a single pathway rather than targeting two parallel pathways.

### RNAi-based screen

To determine whether the male-induced gene expression changes in hermaphrodites functionally contribute to their premature demise, we performed a targeted RNAi-based screen. The specific genes that we included in our screen were chosen because they were upregulated in several datasets: *glp-1* sterile hermaphrodites for long and short interactions (Fig. 1g) and wild-type or feminized individuals mated with males for 2 h^13^ (Extended Data Fig. 1d). We also included several genes that were highly male-enriched (*lys-3*, *C29F7.2*, *T02B5.3* and *T16G1.6*) and that were not significantly enriched (*kgb-1* and *K09C4.5*) as controls to test whether genes that we filtered from our RNA-seq analysis were functionally important for male-induced demise. None of these genes significantly extended hermaphrodite lifespan when knocked down (Supplementary Table 1). As a positive control, we used RNAi knockdown of the male-induced demise gene *utx-1* (ref. ^1^). RNAi treatments were blinded until the last animals died.

For the screen, we performed lifespan assays using a single plate of approximately 35 N2 (wild type) hermaphrodites for each ‘no males’ condition and two plates of approximately 18 N2 (wild type) hermaphrodites and 18 *him-5(e1467)* males for each ‘+ males’ condition (Lifespan assays).

### Classification of RNAi and mutant lifespan results using the Cox proportional hazard model

To test whether there is a significant interaction between a specific gene perturbation and the presence of males for impact on lifespan, we used the Cox proportional hazard model—a statistical method to examine association between survival (lifespan) and other parameters (in this case, gene perturbation and presence of males). The results of this statistical test were interpreted as follows:

1. Hazard ratio < 1 and *P* value < 0.05 (for screen only: *P* value < 0.1): genes that, when knocked down or mutated, exhibit a significant and protective interaction with the presence of males (that is, specific and protective to male-induced demise). This category includes (a) genes that, when knocked down or mutated, only extend lifespan in the presence of males but not in the absence of males (for example, *delm-2* knockdown, *acbp-3* knockdown) and (b) genes that, when knocked down or mutated, extend lifespan to a greater extent in the presence of males than in the absence of males (but still extend lifespan in the absence of males) (for example, triple mutant for *delm-1*, *delm-2* and *acd-1*). This category of genes (male-specific, protective) is denoted by green squares throughout the manuscript.
2. Hazard ratio > or < 1 and *P* value > 0.05 (for screen only: *P* value > 0.1): Genes that, when knocked down or mutated, do not exhibit a significant interaction with the presence of males. This category includes (a) genes that, when knocked down or mutated, have equivalent ability to extend lifespan in the presence and absence of males (for example, *sri-40*, *utx-1*) (this category of genes (male-independent, generally protective) is denoted by yellow squares throughout the manuscript); and (b) genes that when knocked down or mutated have no impact on lifespan in the presence of absence of males (for example, *pud-2.1* knockdown). This category of genes (no effect of lifespan) is denoted by gray squares throughout the manuscript.
3. Hazard ratio > 1 and *P* value < 0.05 (for screen only: *P* value < 0.1): genes that, when knocked down or mutated, exhibit a significant and detrimental interaction with the presence of males. This category includes (a) genes that, when knocked down or mutated, extend lifespan only in the absence of males, but do not extend lifespan in the presence of males (for example, *jmjd-1.2*) and (b) genes that, when knocked down or mutated, extend lifespan to a greater extent in the absence of males than in the presence of males (and extend lifespan in the absence of males) (for example, *daf-2* mutant). This category (male-specific, detrimental) was noted by purple squares.

Thus, this classification based on Cox proportional hazard model yields four categories of genes: (with a *P* value cut-off of 0.1 for the screen and 0.05 for all other lifespan assays):

1. Genes that, when knocked down or mutated, exhibit a significant and protective interaction with the presence of males (that is, specific and protective to male-induced demise) (green squares)
2. Genes that, when knocked down or mutated, do not exhibit a significant interaction with the presence of males (independent of males) (yellow squares)
3. Genes that, when knocked down or mutated, do not exhibit a significant interaction with the presence of males and do not have an impact on lifespan (gray squares)
4. Genes that, when knocked down or mutated, exhibit a significant and detrimental interaction with the presence of males (purple squares)

Note that the tops hits of our screen were tested with additional replicates with around 100 individuals per condition per replicate (Fig. 2m and Supplementary Table 1).

### Mating efficiency

To determine whether a gene knockdown impacts the rate of mating between males and hermaphrodites, we measured mating efficiency using a modified version of a published protocol^44^. Briefly, 2 days before the mating assay, synchronized, L4 hermaphrodites were moved onto dsRNA producing bacteria (individuals were moved to plates randomly and the RNAi knockdown treatment was blinded) to initiate gene knockdown (RNAi knockdown). The day before the mating assay, adult day 1 males (*him-5(e1467))* were fluorescently labeled by culturing them overnight on NGM plates seeded with 100 μl stationary phase OP50-1 and 5 ng μl^–1^ MitoTracker Red CMXRos (Thermo Fisher catalog. no. M7512, resuspended in DMSO (Fisher) at 0.5 mg μl^–1^ and kept at –20 °C in aliquots) at a density of approximately 100 males per 6-cm plate. On the day of the mating assay, 40 fluorescently labeled males and 20 unlabeled hermaphrodites were placed on 6-cm NGM plates seeded with OP50-1 bacteria. Animals were allowed to interact with each other and mate for 2 h at their normal culturing conditions. Following this period, hermaphrodites that had mated successfully were identified based on the presence of fluorescence (male sperm) in their uterus and/or spermatheca using a fluorescent dissecting microscope. The number of mated hermaphrodites was compared with the total number of hermaphrodites per plate (mating efficiency = number of hermaphrodites with male sperm / total number of hermaphrodites).

### CRISPR–Cas9 knockout of *acd-1*

The paralogs *acd-1* and *delm-2* are present in tandem on the same chromosome in the *C. elegans* genome. To generate a double *acd-1* *delm-2* mutant strain, we used CRISPR–Cas9 gene editing as described previously^45^ to knockout *acd-1* in a *delm-2(ok1822)* background (strain RB1523). A CRISPR RNA (crRNA) for *acd-1* was designed using the predesigned Alt-R CRISPR–Cas9 guide RNA tool from IDT. To generate the crRNA:trans-activating crRNA (tracrRNA) duplexes, we used 200 µM Alt-R CRISPR crRNA for *acd-1*, 200 µM tracrRNA and 20 µM crRNA for *dpy-10*, which were annealed for 5 min at 95 °C followed by 5 min at room temperature. Next, 27 µM crRNA:tracrRNA duplex was mixed with 27 µM Cas9-NLS protein (IDT) and incubated for 5 min at room temperature. Finally, the injection mix was assembled with components having the final concentrations of: 17.5 µM Cas9 protein, 17.5 µM crRNA:tracrRNA duplexes and 0.5 µM single-stranded DNA repair template for *dpy-10* (ref. ^46^). The addition of the *dpy-10* crRNA and repair template allows for a rapidly identifiable marker of CRISPR editing that is crossed out of the strain during backcrossing^46^. Young *delm-2(ok1822)* mutant hermaphrodites were injected and left to recover on individual plates. After 3–5 days, plates were screened for rollers or dumpy worms and then subsequently screened for *acd-1* mutations using Sanger sequencing. The resulting strain was backcrossed three times (ABR212, *delm-2(ok1822*) *acd-1(sta6)*). A triple mutant (ABR225) was made by crossing ABR212 with RB1177 (*delm-1(ok1226)*).

Guide and repair template sequences:

*dpy-10* crRNA: GCUACCAUAGGCACCACGAG

*dpy-10* repair template ssDNA: CACTTGAACTTCAATACGGCAAGATGAGAATGACTGGAAACCGTACCGCATGCGGTGCCTATGGTAGCGGAGCTTCACATGGCTTCAGACCAACAGCCTAT

*acd-1* crRNA**:** CATAATGGTGTCGTGTTCCC

### Alignment of *delm-2* RNAi targeting sequence

The *delm-1* and *acd-1* unspliced transcript sequences were downloaded from WormBase (WS275) and aligned to the Ahringer library RNAi construct that targets *delm-2* using Clustal Omega^47^. The pairwise alignments were visualized using JalView^48^.

### Fluorescent reporters

To quantify the mitochondrial UPR induction and FAT-5 and FAT-7 expression, transgenic reporters (Worm strains and maintenance) were used^49,50^. For the mitochondrial UPR, *hsp-6p::GFP* reporters were crossed to the mitochondrial mutants *nuo-6(qm200)* and *isp-1(qm150)* to generate strains ABR213 and ABR214, respectively. Hermaphrodites were synchronized using a timed egg lay and were cultured on RNAi plates seeded with HT115 *E. coli* containing either EV or *delm-2* targeting dsRNA (individuals were selected randomly for each condition in an alternating manner). Starting on adult day 1, synchronized hermaphrodites were randomly selected in an alternating fashion to be either cultured only with other hermaphrodites (around 35 per 6-cm plate) or with males (around 15 hermaphrodites and around 30 adult day 1 *him-5(e1467)* males). After 2 days (adult day 3), hermaphrodites were anaesthetized in M9 (22 mM KH_2_PO_4_, 42 mM Na_2_HPO_4_, 86 mM NaCl and 1mM MgSO_4_) containing 50 mM sodium azide and mounted on 2% agar pads. All images were acquired on a Nikon Eclipse confocal with identical exposure settings for all conditions within a given experiment. Mean fluorescence intensity of the whole worm was quantified in FIJI after summing the fluorescence intensity of all images in a stack. No statistical methods were used to predetermine sample sizes but ours are similar to those reported in previous publications^51^. The quantification results are in Source Data.

### FOXO/DAF-16 localization

To measure nuclear localization of FOXO/DAF-16, we used a fluorescent DAF-16 reporter^52^ (Worm strains and maintenance). Hermaphrodites were synchronized using a timed egg lay on RNAi plates seeded with HT115 *E. coli* containing EV. Starting at adult day 1, synchronized hermaphrodites were randomly selected in an alternating fashion to be either cultured only with other hermaphrodites (around 35 per 6-cm plate) or with males (around 15 hermaphrodites and around 30 adult day 1 *him-5(e1467)* males). After 2 days (adult day 3), half of the hermaphrodites were exposed to a 2-h heat shock at 30 °C by wrapping the plates in Parafilm and submerging in a waterbath. The worms were immediately anaesthetized in M9 (22 mM KH_2_PO_4_, 42 mM Na_2_HPO_4_, 86 mM NaCl and 1 mM MgSO_4_) containing 50 mM sodium azide, mounted on 2% agar pads and imaged. To avoid the stress of anesthesia and mounting to influence DAF-16 localization, worms were imaged within 15 min. All images were acquired on a Nikon Eclipse confocal with identical exposure settings for all conditions within a given experiment. When determining nuclear versus cytoplasmic localization of DAF-16, all images were blinded and individual worms scored as primarily cytoplasmic, primarily nuclear or nuclear and cytoplasmic DAF-16::GFP localization in the anterior intestinal cells.

### Microarrays

To identify the male features involved in the induction of specific genes, we performed microarrays (also described previously^32^). Briefly, *glp-1(e2141)* hermaphrodites were mated with wild-type or *fer-6(hc6)* males starting on day 1 of adulthood for 24 h in a 2:1 male to hermaphrodite ratio. *fer-6(hc6)* males do not transfer sperm but have normal seminal fluid and copulation during mating^3,26^. Next, 200 hermaphrodites were collected on day 2/3 of adulthood. Four biological replicates of wild-type males mated *glp-1* hermaphrodites were collected on day 2 of adulthood, another three replicates were collected on day 3 of adulthood. All six replicates of *fer-6* male-mated *glp-1* hermaphrodites were collected on day 3 of adulthood.

Because male pheromone-induced demise depends on an intact germline^11^, male pheromone-induced expression changes might be missed in our RNA-seq and microarray transcriptomic measurements using *glp-1* (germline-less) animals. Therefore, we identified male pheromone-induced expression changes using wild-type (N2) hermaphrodites. Synchronized late L4 N2 hermaphrodites were picked onto 35-mm plates (control and MCP (conditioned by 30 young *fog-2* males for 2 days)). A total of 30 worms per plate—about 180 worms in total—were used for each biological replicate. Hermaphrodites were transferred onto freshly seeded or MCP every 2 days and collected on day 6 for RNA extraction. Four biological replicates were performed. Interestingly, MCP-induced hermaphrodite gene expression changes that were very similar to the transcriptional response of males to MCP (upregulated genes *P* value = 0.00057 and downregulated genes *P* value = 4.96 × 10^–5^, hypergeometric test)^11^.

RNA was extracted by the heat vortexing method. Two-color Agilent microarrays were used for expression analysis. Significantly differentially expressed gene set were identified using SAM^53^. Additional analysis was performed in R (v.3.5.1) and all R code is publicly available online (https://github.com/brunetlab/Booth-et-al.-2022).

Microarray data can be found in Supplementary Data 5–7 and PUMAdb (http://puma.princeton.edu):

*glp-1* hermaphrodites mated with wild-type males:

https://puma.princeton.edu/cgi-bin/exptsets/review.pl?exptset_no=7345

*glp-1* hermaphrodites mated with *fer-6* males:

https://puma.princeton.edu/cgi-bin/exptsets/review.pl?exptset_no=7346

N2 hermaphrodites on MCP:

https://puma.princeton.edu/cgi-bin/exptsets/review.pl?exptset_no=7351

### Comparison of gene expression results

The transcriptomic experiments (Figs. 1 and 4) were performed by different researchers using different experimental setups (for example, the ratio of males to hermaphrodites) and nematode strains. To compare the RNA-seq and microarray gene expression results, we calculated Pearson’s correlations of the fold changes for the genes measured in the different experiments (Extended Data Fig. 4a), identified the genes that are common between the microarray and RNA-seq results and performed hypergeometric tests (Extended Data Fig. 4b), and compared gene set enrichments (Extended Data Fig. 4c). These comparisons revealed that the RNA-seq and microarray yielded highly similar results at the whole-genome and specific gene levels, highlighting the robustness of the male-induced demise phenomenon and our data.

### Transcription factor binding enrichment

ChIP-Atlas^54^ was used to determine the enrichment of chromatin immunoprecipitation–sequencing (ChIP–seq) peaks at the male sperm, seminal fluid and pheromone regulated genes. These differentially expressed genes were identified by microarrays. The ChIP–seq peak significance threshold was set to 100 (equivalent to a *Q* value < 1 × 10^–10^) and ChIP–seq peaks from the groups ‘TFs and others’ and ‘All cell types’ were used. ChIP–seq peaks within 2 kb^55^ up- and downstream of the transcription start site were considered for each differentially expressed gene. The presence and strength of transcription factor peaks for select genes (Fig. 5d) was also determined using ChIP-Atlas on default settings. We note that these ChIP–seq experiments were performed in whole worms at various developmental stages and under different conditions. Thus, it is important to keep in mind that these transcription factor-gene regulatory connections may not represent the situation in a given adult tissue undergoing male-induced demise. The complete list of enriched transcription factor binding is in Supplementary Data 9.

### Motif analysis

To calculate known and de novo motif enrichment within the promoter regions of differentially expressed genes, we used the Homer^56^ function ‘findMotifs.pl’ with parameters ‘-start -300 -end 300’ (we also tested a larger region with ‘-start -2000 -end 2000’ and found similar results).

To identify whether specific motifs were enriched in the promoter regions of differentially expressed genes, we first downloaded the PSWM files for the FOS, PBX3 and PQM-1 binding motifs from Homer^56^. We used the ChIPSeeker^57^ function ‘getPromoters’ with parameters ‘TxDb=TxDb.Celegans.UCSC.ce11.refGene, upstream=300, downstream=300’ to assign promoters to genes from lists of differentially expressed genes generated from the RNA-seq data with a statistical significance threshold of FDR < 0.05 (we also tested a larger region with ‘upstream=2000, downstream=500’ and found similar results). The resulting bed files were converted to FASTA format with bedtools ‘getfasta’^58^ to be made compatible with the MEME suite AME^59^. The MEME Suite AME^59^ (with default parameters ‘Average odds score’ and ‘Fisher’s exact test’ using shuffled input sequences as the control) was used with to determine statistical enrichment of the chosen motifs. We found that the PQM-1 motif was significantly enriched at the male sperm upregulated (*P* = 3.87 × 10^–22^) and downregulated (*P* = 1.23 × 10^–20^) genes, the male seminal fluid upregulated (*P* = 5.87 × 10^–3^) and downregulated (*P* = 9.8 × 10^–19^) genes and the male pheromone upregulated genes (*P* = 4.93 × 10^–6^).

### Tissue expression predictions

The tissue expression patterns of the male sperm, seminal fluid and pheromone regulated genes were predicted using the webtool https://worm.princeton.edu^30^. The male-induced, differentially expressed genes were identified by microarrays and were used as input. The tissue expression prediction results were clustered and displayed as a heatmap using the R package pheatmap (v.1.0.12). The weighted average expression in a tissue for a gene set was calculated as the mean male-induced expression prediction score divided by the mean expression prediction score for all gene detected by microarray.

### Single-cell RNA-seq reanalysis

To identify cell- and tissue-specific expression patterns for *delm-2* and *acbp-3*, we reanalyzed published single-cell RNA-seq data from larval stage 2 (L2) hermaphrodites^31^. Briefly, the data from the two single-cell experiments were merged and clustered using Seurat v.3.2.3 (refs. ^60,61^) and Harmony (v.1.0 (ref. ^62^)). We performed uniform manifold approximation and projection (UMAP) clustering on the first 35 principal components after performing PCA on the 2,000 most variable genes. Expression of *delm-2* and *acpb-3* was projected onto the UMAP with a maximum cut-off of three. The original, published cell type and tissue identifiers (for example intestine, body wall muscle) were used.

### ORO staining

ORO staining was used to measure neutral lipids in fixed worms. Wild-type hermaphrodites were age-synchronized using a timed egg lay on RNAi plates seeded with EV HT115 *E. coli*. At adult day 1 (day 3 of life), hermaphrodites were placed randomly on either EV RNAi or *delm-2* RNAi and cultured either only with other hermaphrodites or with *him-5(e1467)* adult day 1 males (1:1 ratio). ORO staining was conducted as described^63^, except that 2% paraformaldehyde in PBS was used to fix worms and an overnight incubation with ORO dye (Sigma-Aldrich, dissolved in 60% isopropanol at 5 mg ml^–1^) was used. ORO-stained worms were mounted onto 2% agar pads and imaged at ×10 magnification using a Zeiss AxioSkop 2 Plus. The same exposure settings were used across all conditions within each experiment. Using FIJI image processing software, raw images were subtracted from background, converted to grayscale and inverted. To quantify whole-worm neutral lipid levels, the segmented line tool was used to define a region of interest for the entire worm for each individual and mean ORO intensity measure. Mean intensity values were graphed using Graphpad Prism, and statistical significance was determined using a two-tailed Mann–Whitney test (pairwise comparisons) and two-way analysis of variance (ANOVA). No statistical methods were used to predetermine sample sizes but ours are similar to those reported in previous publications^32,51^. For figures containing straightened worm, unmodified images of representative worms were selected and straightened.

### Statistics and reproducibility

The sample sizes for all lifespan assays were chosen using field standards^41^ and are listed in Supplementary Table 1. For all experiments, nematodes were selected for controls or treatments in a random and alternating manner and were progeny from the same cohort of parents. Whenever possible, the experiments were performed blinded (for example, the RNAi treatment or genotypes without a phenotype that could be discerned by eye). However, in some cases blinding was not possible (for example, comparing the effects of the presence versus absence of males on lifespan). Data were excluded if they failed to meet pre-established quality control criteria. For example, two RNA-seq libraries were excluded before sequencing because they did not meet quality control criteria for insert size distribution and purity. For lifespan assays, worms were scored as censored if they crawled off the media or died due to bagging (internal hatching) or vulval rupture, following field standards. Data from these censored worms were included up until the point of censorship (see Supplementary Table 1 for all data).

In many cases, experiments were repeated by independent investigators or results were verified by an orthogonal method. See, for example, Supplementary Table 1 for a complete list of lifespan assays replicated by independent researchers. In addition, our transcriptomic experiments (RNA-seq and microarrays) were performed by different researchers at different institutes. These experiments show highly similar results.

Lifespan data were plotted as Kaplan–Meier survival curves and pairwise statistical analyses were performed using the logrank (Mantel–Cox) test and the Cox proportional hazard model to model the effect of a gene knockdown or mutation in either the presence or absence of males. The Cox proportional hazard model was also used to test for an interaction between the effects of the presence of males and a specific mutation and RNAi treatment on hermaphrodite lifespan. For the ORO and fluorescence microscopy experiments, conditions were compared by a two-tailed, Mann–Whitney test and two-way ANOVA.

### Reporting summary

Further information on research design is available in the Nature Research Reporting Summary linked to this article.

## Supplementary information

Reporting Summary

Supplementary Data 1 DESeq2 calculated RNA-seq differential expression after removal of male-enriched transcripts.

Supplementary Data 2 Gene set enrichment of the RNA-seq differentially expressed genes (after removal of male-enriched transcripts).

Supplementary Data 3 DESeq2 calculated RNA-seq differential expression.

Supplementary Data 4 Gene set enrichment of the RNA-seq differentially expressed genes.

Supplementary Data 5 Microarray SAM results for WT male-mated hermaphrodites.

Supplementary Data 6 Microarray SAM results for spermless (*fer-6)* male-mated hermaphrodites.

Supplementary Data 7 Microarray SAM results for MCP exposed hermaphrodites.

Supplementary Data 8 Gene set enrichment of the microarray differentially expressed genes.

Supplementary Data 9 Transcription factor ChIP–seq enrichment.

Supplementary Table 1 Lifespan results.

## Data Availability

All RNA-seq reads are available on NCBI Sequence Read Archive (PRJNA642294). Figure 1 is associated with raw data that can be found under this accession code. The results from the microarrays (Fig. 4) are associated with raw data that is available at http://puma.princeton.edu (Methods). In addition, we have included the complete results of the RNA-seq and microarray analyses and the raw data from each worm measured by microscopy (fluorescence and ORO staining) as Supplementary Data and Source Data. The complete list of all lifespan assays (including statistics and number of animals) is presented in Supplementary Table 1.

## Source data

Source Data Fig. 3 Cox Proportional Hazard Model Results and Individual hermaphrodite mitoUPR fluorescence quantification.
Source Data Fig. 7 Individual hermaphrodite microscopy quantification values.
Source Data Extended Data Fig. 2 Cox Proportional Hazard Model Results, mating efficiency results, Individual hermaphrodite mitoUPR fluorescence quantification, DAF-16::GFP localization results.

## Code availability

All code is available on GitHub (https://github.com/brunetlab/Booth-et-al.-2022).
