# Supplementary material for: Males induce premature demise of the opposite sex by multifaceted strategies
Source: Nat Aging. 2022 Sep 16;2(9):809–23. doi: 10.1038/s43587-022-00276-y (PMC10154206; doi:10.1038/s43587-022-00276-y)
Supplement: Supplementary file 1 — Reporting Summary [file 43587_2022_276_MOESM1_ESM.pdf]

nature portfolio

Corresponding author(s): Anne Brunet
Coleen T Murphy

Last updated by author(s): Jul 17, 2022

Reporting Summary

Nature Portfolio wishes to improve the reproducibility of the work that we publish. This form provides structure for consistency and transparency in reporting. For further information on Nature Portfolio policies, see our Editorial Policies and the Editorial Policy Checklist.

Statistics

For all statistical analyses, confirm that the following items are present in the figure legend, table legend, main text, or Methods section.

n/a	Confirmed	
☐	☒	The exact sample size (*n*) for each experimental group/condition, given as a discrete number and unit of measurement
☐	☒	A statement on whether measurements were taken from distinct samples or whether the same sample was measured repeatedly
☐	☒	The statistical test(s) used AND whether they are one- or two-sided<br>*Only common tests should be described solely by name; describe more complex techniques in the Methods section.*
☒	☐	A description of all covariates tested
☐	☒	A description of any assumptions or corrections, such as tests of normality and adjustment for multiple comparisons
☐	☒	A full description of the statistical parameters including central tendency (e.g. means) or other basic estimates (e.g. regression coefficient) AND variation (e.g. standard deviation) or associated estimates of uncertainty (e.g. confidence intervals)
☐	☒	For null hypothesis testing, the test statistic (e.g. *F*, *t*, *r*) with confidence intervals, effect sizes, degrees of freedom and *P* value noted<br>*Give P values as exact values whenever suitable.*
☒	☐	For Bayesian analysis, information on the choice of priors and Markov chain Monte Carlo settings
☒	☐	For hierarchical and complex designs, identification of the appropriate level for tests and full reporting of outcomes
☒	☐	Estimates of effect sizes (e.g. Cohen's *d*, Pearson's *r*), indicating how they were calculated

Our web collection on statistics for biologists contains articles on many of the points above.

Software and code

Policy information about availability of computer code

Data collection	No software was used
Data analysis	Data analysis was performed using R (v3.2.4 or 3.5.1). Key packages used were: STAR (v2.5.4a), DESeq2 (vl.10.1), Biobase (v2.30.0 and v2.42.0), ggplot2 (v3.3.0), pheatmap (vl.0.12), vennerable (v3.l.0.9000), Seurat (v3.2.3), and Harmony (v1.0). Prism v8.4 was used. All custom code used for the study are publicly available on GitHub (https://github.com/brunetlab/Booth-et-al.-2022).

For manuscripts utilizing custom algorithms or software that are central to the research but not yet described in published literature, software must be made available to editors and reviewers. We strongly encourage code deposition in a community repository (e.g. GitHub). See the Nature Portfolio guidelines for submitting code & software for further information.

Data

Policy information about availability of data

All manuscripts must include a data availability statement. This statement should provide the following information, where applicable:

- Accession codes, unique identifiers, or web links for publicly available datasets
- A description of any restrictions on data availability
- For clinical datasets or third party data, please ensure that the statement adheres to our policy

All RNA-seq reads are available on NCBI Sequence Read Archive (PRJNA642294). Figure 1 is associated with raw data which can be found under this accession code. The results from the microarrays (Figure 3) are associated with raw data and these data are available at http://puma.princeton.edu (see Methods). In

addition, we have included the complete results of the RNA-seq and microarray analyses and the raw data for each worm measured with microscopy (fluorescence intensity and Oil Red O staining) as Source Data. The complete list of all lifespan assays (including statistics and number of animals) in presented in Supplementary Table 1.

Human research participants

Policy information about studies involving human research participants and Sex and Gender in Research.

Reporting on sex and gender	n/a
Population characteristics	n/a
Recruitment	n/a
Ethics oversight	n/a

Note that full information on the approval of the study protocol must also be provided in the manuscript.

Field-specific reporting

Please select the one below that is the best fit for your research. If you are not sure, read the appropriate sections before making your selection.

☒ Life sciences ☐ Behavioural & social sciences ☐ Ecological, evolutionary & environmental sciences

For a reference copy of the document with all sections, see nature.com/documents/nr-reporting-summary-flat.pdf

Life sciences study design

All studies must disclose on these points even when the disclosure is negative.

Sample size	The chosen sample size for all lifespan assays was determined using field standards (Lucanic et al. Nature Communications, 2017). The sample sizes for each experimental condition are listed in Supplementary Table 1.
Data exclusions	Data was excluded if it failed to meet pre-established quality control criteria. Specifically, two RNA-seq libraries failed our quality controls (BioAnalyzer quality control for insert size distribution and purity) prior to sequencing and were excluded.<br>For lifespan assays, worms that bagged, ruptured or crawled off of the plate were censored, consistent with field standards. The number of worms censored for each assay is listed in Supplementary Table 1.
Replication	All lifespan replicates are shown and includes results that were replicated independently by different researchers (see Supplementary Table 1 which includes all stats and the identity of the researcher for each experiment). In addition, our transcriptomic experiments (RNA-seq and microarray) were performed by two different researchers at different institutes. These experiments show highly similar results (see Extended Data Fig. 4). All microscopy experiments were performed at least twice and the data from the independent replicates are in Source Data.
Randomization	Worms were split from the same egg lay between control and treatment groups. Both male and hermaphrodite worms were randomly assigned to groups in an alternating and blinded fashion to avoid selection bias. Randomization was performed for the worms used for the RNA-seq, microarrays, microscopy, and lifespan assays.
Blinding	For all RNAi and mutant lifespan assays the identity of the RNAi treatment or mutation was blinded until the last animal died (Fig. 2a-j, 3d-f, i, 4c, d, f, g and Extended Data Fig. 2a-h, 3c-h, 5). Blinding on the basis of the presence or absence of males in lifespan assays is not possible. The RNAi treatments for the worms used in the mating efficiency assay were also blinded (Extended Data Fig. 2i). For Figure 2l and Extended Data Figure 2k, the hermaphrodites were blinded based on treatment when nuclear vs. cytoplasmic localization was determined. For the fluorescence (Figure 2k, 5f, g and Extended Data Figure 2j) and Oil Red O quantification (Figure 5a-e), treatments were not blinded but quantification of the entire worm was performed using FIJI and the experimenter used identical methods across all conditions.

Reporting for specific materials, systems and methods

We require information from authors about some types of materials, experimental systems and methods used in many studies. Here, indicate whether each material, system or method listed is relevant to your study. If you are not sure if a list item applies to your research, read the appropriate section before selecting a response.

Materials & experimental systems

n/a	Involved in the study
☒	☐ Antibodies
☒	☐ Eukaryotic cell lines
☒	☐ Palaeontology and archaeology
☐	☒ Animals and other organisms
☒	☐ Clinical data
☒	☐ Dual use research of concern

Methods

n/a	Involved in the study
☒	☐ ChIP-seq
☒	☐ Flow cytometry
☒	☐ MRI-based neuroimaging

Animals and other research organisms

Policy information about studies involving animals; ARRIVE guidelines recommended for reporting animal research, and Sex and Gender in Research

Laboratory animals	C. elegans hermaphrodite and male worms were used. A complete strain list is available in the Methods section.
Wild animals	n/a
Reporting on sex	This study was performed with hermaphrodites and male C. elegans and the sex of the animals in each experiment is clearly labeled.
Field-collected samples	n/a
Ethics oversight	No ethical approval is required for studies with nematodes.

Note that full information on the approval of the study protocol must also be provided in the manuscript.

March 2021
